# Assessment of genetic variation in Deenanath grass (*Pennisetum pedicellatum* L.) based on morphological trait and molecular markers

**DOI:** 10.3389/fpls.2026.1801895

**Published:** 2026-05-28

**Authors:** Sunil Kumar Verma, Sanjay Kumar Mohanta, Poonam Nawalkar

**Affiliations:** Department of Molecular Biology and Biotechnology, College of Agriculture, Indira Gandhi Krishi Vishwavidyalaya, Raipur, Chhattisgarh, India

**Keywords:** Deenanath grass, diversity, genetic similarity, molecular markers, morphological traits

## Abstract

Deenanath grass is an economically valuable forage crop with a wide range of applications. Crop improvement efforts in this crop had led to the identification, selection, and release of several varieties with desirable traits for yield, drought tolerance, crude protein content, etc. Understanding of genetic variation is a pre-requisite for any crop improvement program. In this study, we examined the genetic variation in 42 local germplasm of Deenanath grass including one check variety using 25 morphological (18 quantitative and 7 qualitative traits) and 17 SSR markers. *D*^2^ analysis revealed the presence of significant diversity distributed in six different clusters. Clustering pattern indicated that out of 42 genotypes, 21 belonged to cluster I, 11 genotypes belonged to cluster II, clusters III and IV consisted of 1 genotype each, and clusters IV and V consisted of 4 genotypes each. The highest intra-cluster distance was observed in cluster I (213.16), which is composed of 21 genotypes. The highest inter-cluster distance (445.15) was found between clusters III and VI. The polymorphic information content (PIC) value for markers ranged from 0.38 to 0.79. The molecular similarity coefficients ranged from 0.43 to 0.87, indicating substantial diversity among genotypes. The maximum PIC and Rp value was obtained for marker bnlg2228, RM 22109 and bnlg 1927, which indicated its informativeness and usefulness, and confirms the reliability of these molecular markers to study the genetic variation of Deenanath grass. Population structure analysis categorized the genotypes into two subpopulations, POP1 (wild type) and POP2 (cultivated type). The most closely related cultivars were RDC-4, RDC-27, RDC-20, and RDC-21, with the highest similarity index (0.87). The most distantly related cultivars were RDC-12 and RDC-1, with the lowest similarity index (0.47). Genotypes RDC-20, RDC-21, and RDC-6 are recommended as promising parental material for future breeding programs aimed at enhancing both productivity and adaptability in Deenanath grass.

## Introduction

*Pennisetum pedicellatum* Trin. (2n=2x=36), commonly known as Deenanath grass, is an indigenous grass of Ethiopia belonging to the Poaceae family. This species is broadly distributed across different ecological regions, mainly tropical and subtropical zones worldwide. It is particularly common in northern Australia, Southeast Asia, and throughout Africa from west to east. The meaning of Deenanath grass is “friend of the poor”. In India, it occurs widely across several states, mainly Andhra Pradesh, Bihar, Uttar Pradesh, Chhattisgarh, Jharkhand, Karnataka, Maharashtra, Andaman and Nicobar Islands, Odisha, and West Bengal. It is primarily found in disturbed places, along roadsides, in recently cleared ground, and in regions with an annual rainfall between 500 and 1,500 mm, a 4- to 6-month wet season, and typical daytime temperatures between 30 and 35 °C. It exhibits remarkable adaptability and has gained significant attention for its potential as forage or hay, an erosion control agent, and turfgrass species. *P. pedicellatum* Trin. is a prolific tillering annual grass with a high leaf: stem ratio and a low oxalate content. It is a fast-growing, lush, green, and thin-stemmed grass that thrives in eroded, poor soils (Mukherjee et al., 1982). When harvested 60 to 65 days after sowing, it contained high crude protein (9.06%) and crude fat (2.55%), but low crude fiber (28.95%) levels (Khan et al., 1995). It was sown to stop soil erosion and enhance the soil’s physicochemical characteristics. *P. pedicellatum* was also considered as an important source for higher levels of downy mildew resistance (Singh and Navi, 2000).

The continual genetic degradation, which is mostly caused by contemporary agricultural practices, concurrent natural disasters (such as droughts, floods, and fire dangers), human settlements, overgrazing, etc., poses a severe threat to the genetic diversity collected over generations. According to Jarvis et al. (2008), climate change is anticipated to pose an additional hazard to agricultural biodiversity by accelerating the genetic extinction of landraces and endangering wild species, particularly crop wild relatives. Additionally, characterization and evaluation are essential for determining the potential and actual value of germplasm that has been gathered. Therefore, Deenanath grass diversity was gathered in wild habitats from uncharted regions of Chhattisgarh and assessed for utilization in future *Pennisetum* enhancement initiatives using sustainable means in order to stop genetic degradation.

The quantification of the degree of divergence present in the population is of paramount importance in identifying diverse genotypes for recombination breeding programs. Mahalanobis *D*^2^ statistics is a powerful tool for quantifying genetic divergence in germplasm collections with respect to characters considered together.

The genomic diversity is defined as diversity at several DNA loci within an individual (Bhandari et al., 2017). Hence, a study of molecular genetic variation in alignment with the observed agro-morphological traits gives a broad and substantial understanding of diversity for the crop breeders. In case of Deenanath grass, the agro-morphology of only few of the improved varieties is well studied in different environments. However, many of these traits are unstable and show intra-varietal variation in different environments due to genotype × environment interactions (Anandaraj et al., 2014) decreasing their utility as markers and are inefficient to apprehend the real genetic diversity. The DNA sequence-based markers are comparatively stable and not affected by a diverse environment (Nadeem et al., 2018). These are based on polymorphisms at the level of DNA sequence. Hence, molecular characterization would help us to better understand not only the observed agro-morphological diversity but also the real diversity at the DNA level.

Furthermore, an in-depth understanding of the species’ phenotypic and genetic diversity can contribute to the development of improved management strategies for *P. pedicellatum* ecosystems. It enables the identification of ecotypes or varieties with desirable traits, such as enhanced biomass production, drought tolerance, or nutrient utilization efficiency. Such information can facilitate the selection and propagation of superior genotypes for cultivation purposes, thereby promoting sustainable agriculture and land management practices.

## Materials and methods

### Plant material

**Figure 1:**
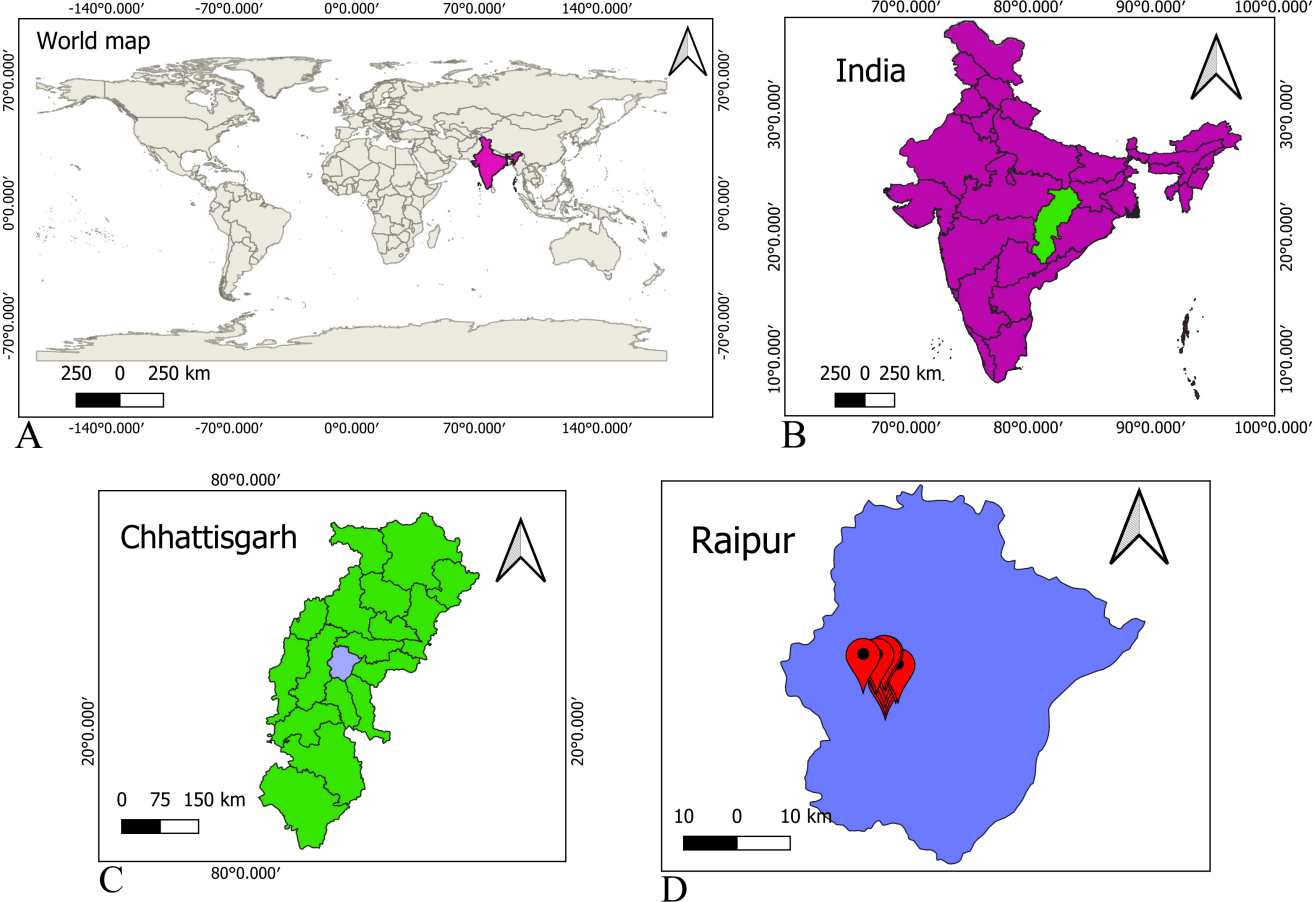
Site of collection of Deenanath grass. **(A)** India in World map, **(B)** Chhattisgarh in India map, **(C)** Raipur in Chhattisgarh map, and **(D)** Collection site (Dharsiwa) in Raipur map.

For morphological and molecular characterization, 42 local germplasm including one check were used for the study. Excluding checks, all 41 germplasm were collected from different villages of Dharsiwa block under the Raipur district of Chhattisgarh, India during *Kharif* 2020 and 2021 (**Table 1**; **Figure 1**). The experiment was conducted at the Instructional cum Research Farm, and the molecular work was done in the Marker Assisted Selection laboratory at the Department of Molecular Biology and Biotechnology, College of Agriculture, Indira Gandhi Krishi Vishwavidyalaya, Raipur (C.G.) during *Kharif* 2022. Seeds of collected germplasm (41 accessions) and one check Bundel Deenanath-2 (BD-2) were grown in the field in 4 × 1.8 m^2^ plots (6 rows of 4m) in a randomized complete block design (RCBD) with two replications. In each plot, there are six rows of 4m at a standard spacing of 30cm, and there is a gap of 1m among all the plots. The recommended practices were followed to raise a healthy crop. The crop was cultivated under rainfed conditions and was handled in accordance with regular agricultural procedures.

**Table 1:** List of genotypes used in present study.

S. no.	Name of genotype	Village	Place of collection			Country	Latitude (N)	Longitude (E)	Altitude (m)	Date of collection
			Block	District	State					
1	RDC-1	Serikhedi, Jora	Dharsiwa	Raipur	C.G.	India	21.234113	81.718548	290	02/11/2021
2	RDC-1b	Serikhedi, Jora	Dharsiwa	Raipur	C.G.	India	21.234109	81.718645	290	03/11/2020
3	RDC-1c	Serikhedi, Jora	Dharsiwa	Raipur	C.G.	India	21.234109	81.718645	290	03/11/2020
4	RDC-2	Serikhedi, Jora	Dharsiwa	Raipur	C.G.	India	21.234150	81.718661	290	02/11/2021
5	RDC-3	Serikhedi, Jora	Dharsiwa	Raipur	C.G.	India	21.233267	81.718577	290	02/11/2020
6	RDC-4	Serikhedi, Jora	Dharsiwa	Raipur	C.G.	India	21.234158	81.718683	290	02/11/2020
7	RDC-4b	Serikhedi, Jora	Dharsiwa	Raipur	C.G.	India	21.234158	81.718683	290	02/11/2021
8	RDC-5	Serikhedi, Jora	Dharsiwa	Raipur	C.G.	India	21.234238	81.717592	290	03/11/2020
9	RDC-6	Serikhedi, Jora	Dharsiwa	Raipur	C.G.	India	21.234791	81.718700	290	02/11/2021
10	RDC-6b	Serikhedi, Jora	Dharsiwa	Raipur	C.G.	India	21.234791	81.718700	290	13/11/2021
11	RDC-7	Serikhedi	Dharsiwa	Raipur	C.G.	India	21.232259	81.718590	286	13/11/2021
12	RDC-8	Serikhedi	Dharsiwa	Raipur	C.G.	India	21.232409	81.719384	286	13/11/2021
13	RDC-9	Serikhedi	Dharsiwa	Raipur	C.G.	India	21.232409	81.719384	286	13/11/2021
14	RDC-10	Serikhedi, Jora	Dharsiwa	Raipur	C.G.	India	21.234230	81.717610	290	13/11/2021
15	RDC-10b	Serikhedi, Jora	Dharsiwa	Raipur	C.G.	India	21.234230	81.717610	290	13/11/2021
16	RDC-11	Serikhedi, Jora	Dharsiwa	Raipur	C.G.	India	21.234230	81.717610	290	03/11/2020
17	RDC-12	Serikhedi, Jora	Dharsiwa	Raipur	C.G.	India	21.234146	81.718400	290	25/11/2021
18	RDC-13	Serikhedi	Dharsiwa	Raipur	C.G.	India	21.231502	81.718331	286	03/11/2020
19	RDC-14	Serikhedi, Jora	Dharsiwa	Raipur	C.G.	India	21.234164	81.718103	286	03/11/2020
20	RDC-15	Serikhedi, Jora	Dharsiwa	Raipur	C.G.	India	21.234164	81.718103	286	03/11/2020
21	RDC-16	Serikhedi	Dharsiwa	Raipur	C.G.	India	21.231165	81.718302	286	03/11/2020
22	RDC-16b	Serikhedi	Dharsiwa	Raipur	C.G.	India	21.231165	81.718302	286	03/11/2020
23	RDC-17	Serikhedi, Jora	Dharsiwa	Raipur	C.G.	India	21.234280	81.717598	290	03/11/2020
24	RDC-18	Serikhedi, Jora	Dharsiwa	Raipur	C.G.	India	21.234113	81.718548	290	03/11/2020
25	RDC-19	Serikhedi, Jora	Dharsiwa	Raipur	C.G.	India	21.234113	81.718548	290	03/11/2020
26	RDC-20	Serikhedi	Dharsiwa	Raipur	C.G.	India	21.233963	81.718787	286	03/11/2020
27	RDC-21	Serikhedi, Jora	Dharsiwa	Raipur	C.G.	India	21.233267	81.718577	290	03/11/2020
28	RDC-22	Serikhedi, Jora	Dharsiwa	Raipur	C.G.	India	21.232744	81.718466	290	03/11/2020
29	RDC-23	Serikhedi	Dharsiwa	Raipur	C.G.	India	21.231965	81.718497	286	13/11/2021
30	RDC-24	Serikhedi	Dharsiwa	Raipur	C.G.	India	21.231965	81.718497	286	13/11/2021
31	RDC-25	Serikhedi, Jora	Dharsiwa	Raipur	C.G.	India	21.231763	81.718325	290	13/11/2021
32	RDC-26	Serikhedi	Dharsiwa	Raipur	C.G.	India	21.231528	81.718328	286	13/11/2021
33	RDC-27	Serikhedi	Dharsiwa	Raipur	C.G.	India	21.230950	81.718296	286	03/11/2020
34	RDC-28	Serikhedi	Dharsiwa	Raipur	C.G.	India	21.230535	81.718245	286	13/11/2021
35	RDC-29	Serikhedi	Dharsiwa	Raipur	C.G.	India	21.230200	81.718188	286	13/11/2021
36	RDC-30	Serikhedi	Dharsiwa	Raipur	C.G.	India	21.232153	81.718328	286	13/11/2021
37	RDC-31	Serikhedi	Dharsiwa	Raipur	C.G.	India	21.231733	81.718462	286	13/11/2021
38	RDC-32	Serikhedi	Dharsiwa	Raipur	C.G.	India	21.232158	81.718191	286	13/11/2021
39	RDC-33	Serikhedi	Dharsiwa	Raipur	C.G.	India	21.234058	81.718870	286	13/11/2021
40	RDC-34	Purena, Jora	Dharsiwa	Raipur	C.G.	India	21.236118	81.702680	291	13/11/2021
41	RDC-35	Serikhedi	Dharsiwa	Raipur	C.G.	India	21.230200	81.718188	286	14/11/2021
42	BD-2	Procured from IGFRI, Jhansi								2021

These local genotypes, except check, were collected from the Dharsiwa Block of Raipur district at the C.G. State of India.

### Data collection and statistical analysis

Data were recorded on seven qualitative traits, namely, early plant vigor, plant growth habit, culm color, branching pattern, leaf hairiness, spikelet color, and biotic stress susceptibility, and on 18 quantitative traits, i.e., plant height (cm), days to 50% flowering, days to maturity, number of nodes per plant, numbers of leaf per plant, leaf length (cm), leaf width (cm), leaf: stem ratio, panicle width (cm), panicle length (cm), number of spikelets per panicle, number of tillers per plant, dry matter yield (q/ha), green fodder yield (q/ha), crude protein yield (q/ha), dry matter %, moisture content (%), and crude protein content (%). Data on qualitative and quantitative traits were recorded on five representative plants except the yield attributing traits. Then, multivariate hierarchical clustering was performed with the means of all characters for the collected local germplasm under study to obtain the overall pattern of morphological diversity of these varieties.

### Genetic divergence by Mahalanobis *D*^2^ statistics

Genetic divergence amongst various genotypes is evaluated on the basis of the estimated *inter se* genetic distances amongst the genotypes. The *D*^2^ statistics of Mahalanobis (1928) is a highly effective tool for measuring the genetic distance between genotypes based on allelic frequencies at specific loci. Moreover, genetic similarity is examined as the opposite of genetic divergence, representing the range of gene similarity observed among the genotypes under consideration.

In the existing investigation, genetic divergence was studied based on Mahalanobis’ generalized distance as suggested by Rao (1952). Actual variable means were converted to un-correlated variables by the pivotal condensation method of inversion matrix.

The *D*^2^ values between the genotypes were achieved as the sum of squares of differences of the values of the equivalent converted variables.

For each pair of combination, the mean deviation, *i.e.*, *di* = *Yi*^1^ – *Yi*^2^, where *Yi* signifies the converted variables (*i*=1, 2, 3, 4, 5 … *p*), was calculated and *D*^2^ was then calculated as the sum of the squares of those deviations, i.e.,


$$D^{2}=\sum {\left(Yi^{1}-Yi^{2}\right)}^{2}$$

where *p* = number of characters.

The importance of *D*^2^ values was tested by treating them as chi-square (χ^2^) at “*p*” degrees of freedom, where *p* is the number of characters reflected.

### Grouping of genotypes by Tocher’s method

Tocher’s approach, introduced by Rao in 1952, is used to cluster genotypes. This method involves arranging the *D*^2^ values of all combinations of genotypes in ascending order. Genotypes with smaller *D*^2^ values are grouped into clusters, indicating their higher similarity compared to those in separate clusters. The procedure calculates inter- and intra-cluster distances and establishes connections based on these measures.

### Average intra-cluster distance

The intra-cluster distances were calculated as


$$\sum Di^{2}/n$$

Where Σ*Di*^2^ is the sum of distance between all possible combinations of (*n*) genotypes included in a cluster. *n* = number of genotypes included in the cluster.

### Average inter-cluster distance

The procedure followed for calculating the inter-cluster distances was first to measure the distance between clusters I and II, between clusters I and III, between clusters I and IV, and so on; likewise, the clusters were taken one by one and the 28 distances from other clusters were calculated. The average inter-cluster distances were then calculated as:


$$\sum Di^{2}/n_{i}n_{j}$$

where *n_i_* = number of genotypes in cluster *i* and *n_j_* = number of populations in cluster *j*.

The intra- and inter-cluster distance (*D*) values were obtained by taking the square root of average *D*^2^ values of the respective genotypes.

### Morphological characterization

Seven morphological traits were recorded to access the diversity among Deenanath grass germplasm (**Table 2**). The early growth habit was recorded at 30 days after sowing by visual assessment of individual genotype, i.e., poor, good, or very good. The plant habit of growth, i.e., erect, decumbent, or prostrate, was recorded at the flowering initiation stage by visual assessment through a single observation of a group of plants or parts of plants. Culm color was recorded at the stage of 50% flowering; the visual assessment of a plant group allowed the observation and documentation of various culm colors, including purple, green, reddish, or pale green. The branching pattern of plants was categorized as absent, low, medium, and high, through visual assessment of individual plants when they reach the 50% flowering stage. During the period of 50% flowering, the visibility of hairs on leaf surfaces was observed, and their density was recorded, categorized as glaborous, sparse, intermediate, and dense. At the stage of nearing maturity, the color of the spikelet was observed through visual assessment of individual genotypes and classified into various categories, including creamy white, light brown, dark brown, light purple, and dark purple. The incidence of significant diseases was documented during the 50% flowering stage, using a scale ranging from 1 to 9 to assess the extent of infestation. A dendrogram and similarity coefficients were generated using the unweighted pair group method with arithmetic mean (UPGMA) in NTSYS-pc version 2.02e (Rohlf, 2000). Here, the SIMQUAL program was utilized in order to evaluate the SM similarity coefficient.

**Table 2:** Description of qualitative characters of Deenanath grass.

S. no.	Characters	Categories	Scores
1	Early growth habit	Poor	1
		Good	2
		Very good	3
2	Plant growth habit	Erect	1
		Decumbent	2
		Prostrate	3
3	Culm color	Pale green	1
		Green	2
		Purple	3
		Reddish	4
4	Branching	Absent	1
		Low	2
		Medium	3
		High	4
5	Leaf hairiness	Glaborous	0
		Sparse	1
		Intermediate	2
		Dense	3
6	Spikelet color	Light brown	1
		Dark brown	2
		Light purple	3
		Dark purple	4
		Creamy white	5
7	Biotic stress susceptibility	Very low	1
		Low	3
		Intermediate	5
		High	7
		Very high	9

### Molecular characterization

#### DNA extraction

For molecular characterization, we isolated DNA from young leaves of all these 42 local germplasm including one check variety, as per the CTAB-based modified protocol (Doyle and Doyle, 1987). DNA concentrations were estimated using a Nanodrop spectrophotometer (Thermo Scientific, Nanodrop 2000c). The DNA of each sample was diluted to a final concentration of 40 ng/µL for PCR analysis.

#### PCR and electrophoresis

We have utilized a total of 17 SSR primers for the study of molecular genetic variation (**Table 3**). PCR amplification was performed with 1.5 µL of template genomic DNA (40 ng/µL), 1 µL of dNTPs (1mM each), 1 µL of PCR buffer including 15 mM MgCl_2_, 0.5 µL (5 pM) of each forward and reverse primer, 0.25 µL of Taq polymerase (1 U/µL), and 5.25 µL of nuclease-free water in a final reaction volume of 10 µL. The conditions for PCR were as follows: 94 °C for 5min (initial denaturation); 94 °C for 1 min, 55 °C annealing temperature for 1 min (depending on the primer used), and extension at 72 °C for 1 min all repeated for 35 cycles; and a final extension at 72 °C for 7 min. The obtained PCR amplicons were loaded onto a 5% PAGE gel along with a 100-bp DNA ladder as band size reference, and electrophoresis was carried out. The gel was stained with ethidium bromide. The gel was then visualized for the separated PCR products using a gel documentation system (Gel Doc TM XR+, BIORAD, USA).

**Table 3:** Details of the 17 microsatellite primers used in present study.

S. no.	Marker name	Forward/reverse	Sequence 5′ to 3′	Annealing temp. (°C)
1.	phi 364545	Forward primer Reverse primer	TAAGCAAAGCAAGGCAACC TCGCCTCACTCTCACACTCC	55
2.	bnlg 1927	Forward primer Reverse primer	TTTTTTTGTAAGCGATCCGG GATGAATCTGCGTCCGTCTT	55
3.	bnlg 2109	Forward primer Reverse primer	GAAGTGTGATCACTGTAACC TACAGTGGACGGCGAAGTCG	55
4.	bnlg 1194	Forward primer Reverse primer	GCGTTATTAAGGCAAGCTGC ACGTGAAGCAGAGGATCCAT	55
5.	RM 25	Forward primer Reverse primer	GGAAAGAATGATCTTTTCATGG CTACCATCAAAACCAATGTTC	55
6.	RM 125	Forward primer Reverse primer	ATCAGCAGCCATGGCAGCGACC AGGGGATCATGTGCCGAAGGCC	55
7.	RM 154	Forward primer Reverse primer	GAAACCACCACACCTCACCG CCGTAGACCTTCTTGAAGTAG	55
8.	RM 277	Forward primer Reverse primer	CTCAAGCTTAGCTGCTGCTG ACTGTGAGATTGACTGACAGTGG	55
9.	RM 28153	Forward primer Reverse primer	ACCTCGCGTTATTAGGTACCC GAGATACGCCAACGAGATAC	57
10.	RM 26213	Forward primer Reverse primer	GCCACAGGAGACAGCAAGAACC CGATCCAATTCCAGCCTAGATAGC	55
11.	RM 13	Forward primer Reverse primer	TCCAACATGGCAAGAGAGAG GGTGGCATTCGATTCCAG	55
12.	RM 22109	Forward primer Reverse primer	CACGACGACGACGAGCAGCA GCTCGAGGGAGAGCGACCTG	55
13.	RM 21625	Forward primer Reverse primer	TCTCCTCTTCCCCCGATC ATAGCGGGCGAGGCTTAG	55
14.	RM 307	Forward primer Reverse primer	GTACTACCGACCTACCGTTCAC CTGCTATGCATGAACTGCTC	55
15.	RM 6054	Forward primer Reverse primer	CCCTCCGTACGGATACACAC CTCTTCGGCTTCATCTCCTC	55
16.	bnlg 2228	Forward primer Reverse primer	GCAGCAATCGACACGAGATA CTTGGATCGCACTCCGTC	55
17.	bnlg 1176a	Forward primer Reverse primer	ACTCCTCAAAACCTAGGTGACA CACCGATGATGGTGAGTACG	55

Source: https://www.gramene.org/ (RM Primers), https://www.maizegdb.org/ (bnlg, phi primers).

### Population structure analysis

The genetic structure (*Q*) prediction and population clustering were performed using the STRUCTURE v 2.3.4 software (Pritchard et al., 2000). In structure analysis, the parameters were set as follows: number of individual, 42; number of loci, 46; number of ploidies, 2; and missing value, −999. The optimum number of populations (*K*) was selected with a burn-in period of 100,000 steps followed by 100,000 Monte Carlo Markov Chain (MCMC) replicates. The range of genetic clusters was set from *K* =1 to *K* =10, and each *K* value was replicated five times. The web interface version of the Structure Selector (https://lmme.ac.cn/StructureSelector/) tool was used to calculate the final population structure. To avoid possible spurious associations, the *Q* (population structure) model was used to account for the population structure and relatedness of individuals among the 42 Deenanath grass genotypes.

### Genetic diversity analysis

The bands obtained after genotyping were scored in a binary format (1 for present, 0 for absent). The polymorphic information content (PIC) of the primers were hence calculated by the formula: $PIC=1-\sum_{i=1}^{n}p_{i}^{2}$ where *p_i_* is the frequency of the *i*th allele of that locus and is the frequency of the null allele (Botstein et al., 1980).

Effective multiplex ratio (EMR) was computed using the formula EMR = n*β, where *n* refers to the average fragment number generated by species to a specific system marker (multiplex ratio) and the computation of parameter β based on the number of polymorphic loci (PB) and non-polymorphic loci (MB), using the formula β = PB/(PB + MB). The marker index (MI) for every primer/marker was determined by multiplying the PIC and EMR (Varshney et al., 2007); MI = EMR × PIC. The resolving power (Rp) of every primer/marker was computed as (Prevost and Wilkinson, 1999): Rp = Σ Ib, where Ib represents the informative fragments. The Ib can be represented on a scale of 0/1 by the following formula: Ib = 1 − (2 *|0.5 − *p_i_*|), where *p_i_* is the proportion of accessions containing the *i*th band.

A dendrogram and similarity coefficients were generated using the UPGMA in NTSYS-pc version 2.02e (Rohlf, 2000). Here, the SIMQUAL program was utilized in order to evaluate the SM similarity coefficient.

## Results

**Figure 2:**
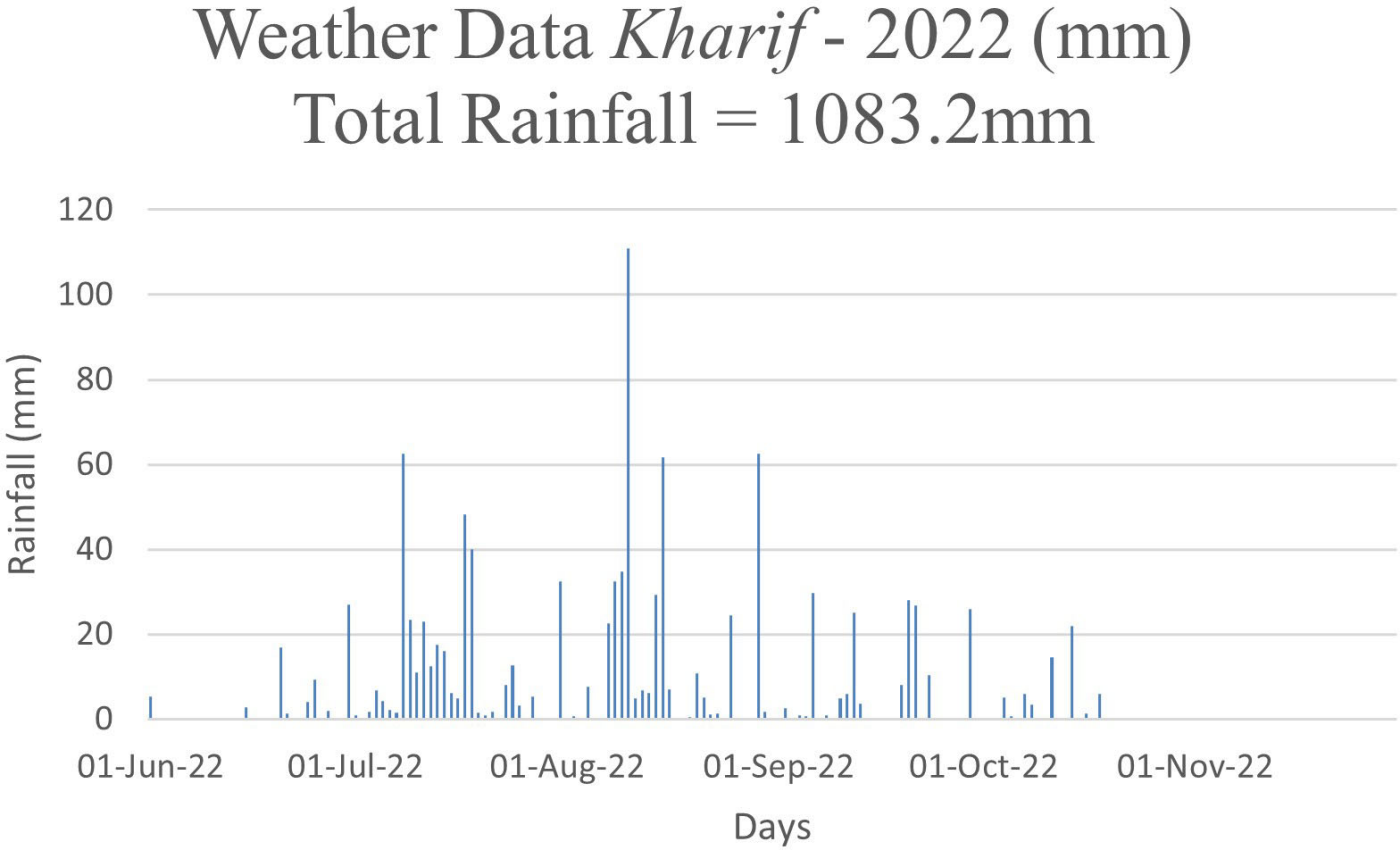
Daily rainfall data during *Kharif* 2022.

During the crop growth period, 1,083.20 mm of rainfall was received, which is sufficient for crop growth. Maximum rainfall was received during August and no rainfall was received in November. During August, a maximum of 111.00mm of rainfall was received in a day (10 August 2022). There has been no rainfall in 108 days. A 43-day break in rainfall was observed from 19 October 2022 (**Figure 2**).

The combination of methods, i.e., cluster analysis based on morphological characterization and the UPGMA dendrogram based on molecular characterization, revealed both the phenotypic and molecular variation among the Deenanath germplasm. This has helped us to understand the pattern of variation, the level of genetic similarity, and the genetic divergence among them.

### Genetic diversity using Mahalanobis *D*^2^ analysis

**Figure 3:**
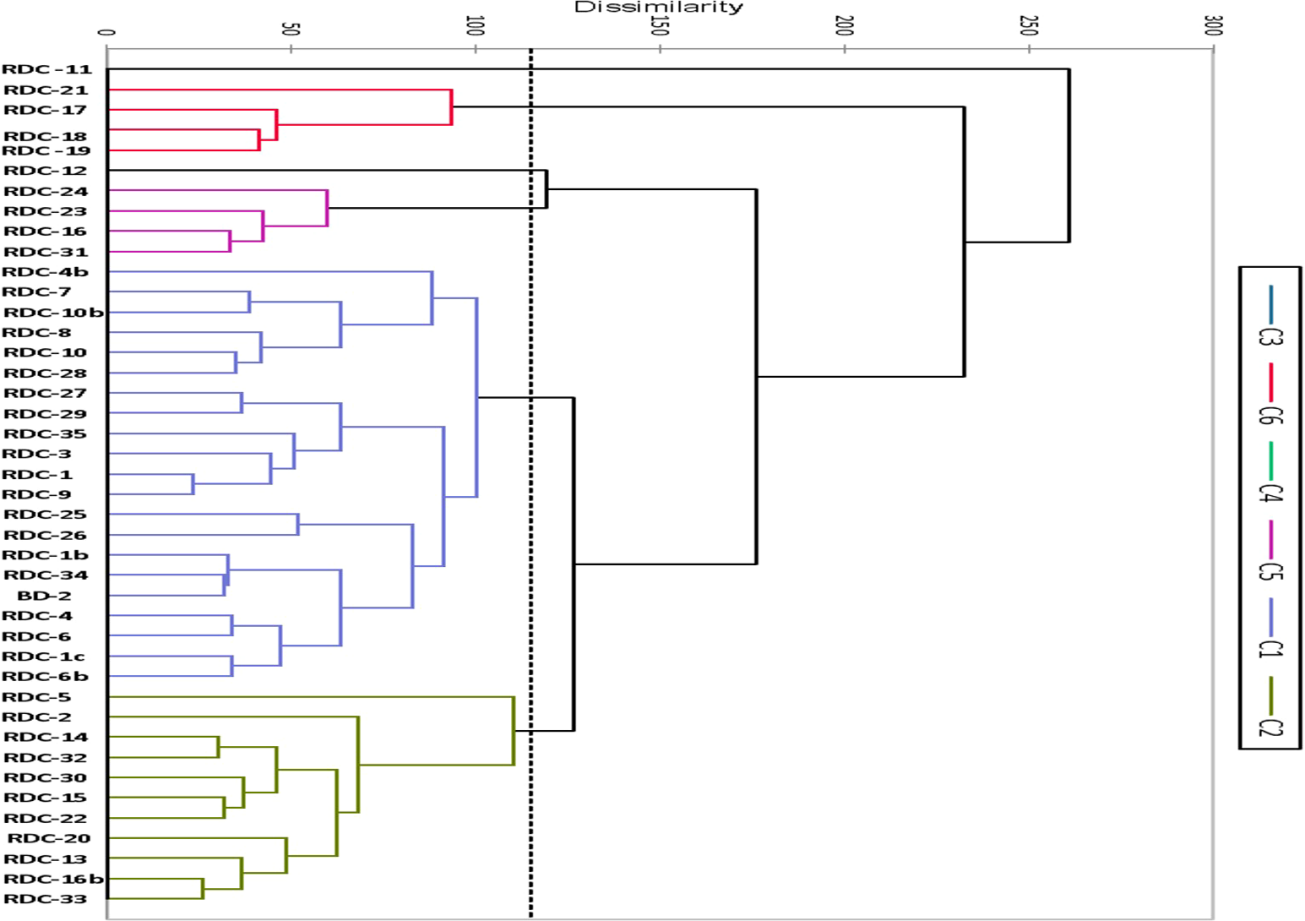
Dendrogram showing the distribution of Deenanath grass genotypes.

The χ^2^ test was used to analyze the “V” statistic, which showed a significant difference in means among the 18 traits studied and between different populations. Consequently, further analysis was conducted using *D*^2^ values to examine genetic divergence. *D*^2^ statistics provide a numerical method for measuring genetic diversity in the germplasm. The intra- and inter-cluster distances, represented by *D*^2^ values, are presented in **Table 4**. **Figure 3** illustrates the diagrammatic representation of clusters, including the intra- and inter-cluster *D*^2^ values.

**Table 4:** Average intra- and inter-cluster distance *D*^2^ values among six clusters of 42 genotypes of Deenanath grass.

	Cluster I	Cluster II	Cluster III	Cluster IV	Cluster V	Cluster VI
Cluster I	**213.16**	107.28	186.02	268.78	163.30	260.51
Cluster II		**151.75**	291.48	224.75	114.23	158.50
Cluster III			**0.00**	387.62	311.85	445.15
Cluster IV				**0.00**	115.52	287.80
Cluster V					**134.15**	216.46
Cluster VI						**101.35**

Bold values indicate the intra-cluster distance.

Cluster mean values for 18 different traits are presented in the **Table 5**. Notably, the extreme mean values for these traits were observed in different clusters. Cluster IV exhibited the highest mean values for number of nodes per plant, leaf blade width (cm), panicle width (cm), number of spikelets per panicle, and moisture content (%). Cluster V had the highest mean values for days to 50% flowering. Cluster III displayed the highest mean values for plant height (cm), days to maturity, GFY (q/ha), DMY (q/ha), number of leaves per plant, leaf blade length (cm), number of tillers per plant crude protein%, and CPY (q/ha). Clusters II and I had the highest mean values for dry matter (%) and leaf: stem ratio, respectively. In contrast, cluster VI exhibited the lowest mean values for plant height, GFY (q/ha), DMY (q/ha), number of leaves per plant, leaf blade length (cm), panicle length (cm), and no. of spikelets per panicle. Similarly, cluster II displayed the lowest values for days to 50% flowering, days to maturity, and moisture content (%), while cluster V had the lowest mean value for no. of nodes per plant, panicle width (cm), and number of tillers per plant. Cluster IV exhibited the lowest mean values for leaf: stem ratio, crude protein (%), dry matter (%), and CPY (q/ha). Finally, the lowest mean value for leaf length was observed in cluster III.

**Table 5:** Cluster mean values for 18 different characters in Deenanath grass.

Traits	Cluster I	Cluster II	Cluster III	Cluster IV	Cluster V	Cluster VI	General mean
Plant height (cm)	191.25	188.65	216.38**	205.82	191.58	159.71*	192.23
Days to 50% flowering	97.71	96.68*	101.00	100.50	102.00**	97.50	99.23
Days to maturity	134.43	130.68*	154.00**	153.50	130.75	141.13	140.75
Green fodder yield (q/ha)	477.02	371.84	652.78**	333.33	365.45	230.90*	405.22
Dry matter yield (q/ha)	125.68	105.63	169.48**	77.53	103.16	59.93*	106.90
No. of nodes per plant	7.27	6.92	7.40	7.80**	6.50*	6.85	7.12
No. of leaves per plant	14.76	15.01	16.60**	14.80	15.10	11.23*	14.58
Leaf blade length (cm)	66.15	68.21	78.19**	66.23	72.10	64.50*	69.23
Leaf blade width (cm)	1.97	1.91	1.86*	2.07**	1.94	1.95	1.95
Panicle length (cm)	13.01	12.55	14.09**	13.99	11.39	11.14*	12.69
Panicle width (cm)	1.06	1.09	1.16	1.83**	0.89*	1.01	1.17
No. of spikelets per panicle	185.14	188.15	207.60	405.70**	301.75	141.70*	238.34
No. of tillers per plant	29.06	29.66	34.30**	27.40	23.15*	27.58	28.53
Leaf:stem	0.38**	0.32	0.31	0.27*	0.30	0.36	0.32
Crude protein %	6.60	6.53	9.91**	5.02*	7.24	6.67	6.99
Moisture content %	73.54	71.54*	74.04	76.74**	71.80	74.45	73.69
Dry matter %	26.46	28.46**	25.96	23.26*	28.20	25.55	26.31
Crude protein yield (q/ha)	8.30	6.81	16.79**	3.89*	7.46	3.69	7.82

**Indicates the highest cluster mean value of the respective trait, *indicates the lowest mean value.

Forty-two Deenanath grass genotypes were grouped into six different clusters based on the *inter se* genetic distances. Grouping of 42 genotypes of Deenanath grass into six clusters is represented in **Table 6**. Clustering pattern indicated that 21 out of 42 genotypes belonged to the same cluster, i.e., cluster I. On the other hand, 11 genotypes belong to cluster II, clusters III and IV consist of 1 genotype each, and clusters IV and V consist of 4 genotypes each. The dendrogram of 42 genotypes of Deenanath grass is represented in **Figure 3**.

**Table 6:** Clustering details of 42 Deenanath grass genotypes based on *D*^2^ statistics.

Cluster	No. of genotypes	Genotype details
I	21	RDC-1, RDC-1b, RDC-1c, RDC-3, RDC-4, RDC-4b, RDC-6, RDC-6b, RDC-7, RDC-8, RDC-9, RDC-10, RDC-10b, RDC-25, RDC-26, RDC-27, RDC-28, RDC-29, RDC-34, RDC-35, BD-2
II	11	RDC-2, RDC-5, RDC-13, RDC-14, RDC-15, RDC-16b, RDC-20, RDC-22, RDC-30, RDC-32, RDC-33
III	1	RDC-11
IV	1	RDC-12
V	4	RDC-16, RDC-23, RDC-24, RDC-31
VI	4	RDC-17, RDC-18, RDC-19, RDC-21

The highest intra-cluster distance was observed in cluster I (213.16), which is composed of 21 genotypes. The highest inter-cluster distance (445.15) was found between clusters III and VI followed by clusters III and IV (387.62), clusters III and V (311.85), and clusters II and III (291.48). The smallest inter-cluster distance (107.28) was observed between clusters I and II followed by clusters II and V (114.23) and clusters IV and V (115.52). The maximum inter-cluster distance was observed between clusters III and VI, which represented high diversity between the genotypes of these clusters, which can be used in the breeding program. In contrast to this, the minimum inter-cluster distance was seen between clusters I and II, which depicted that the genotypes belonging to these clusters are less diverse.

### Morphological characterization

The early growth vigor of 7 out of 42 genotypes of Deenanath grass was found to be inadequate, while 26 genotypes exhibited satisfactory growth and 9 genotypes demonstrated excellent growth.

In the present study, 42 Deenanath grass genotypes were examined to determine their plant growth habit trait, which was classified into three categories: erect (27 genotypes), decumbent (4 genotypes), and prostrate (11 genotypes).

The culm color of 42 genotypes was visually assessed, resulting in observations of varying shades such as pale green, green, purple, and reddish. Among the genotypes, 6 exhibited a pale green color, 14 displayed a green color, and 22 showed a purple appearance.

Among the total of 42 genotypes observed, the majority of them, specifically 26 genotypes, exhibited lower levels of branching. In contrast, 13 genotypes displayed moderate branching, while only 3 genotypes exhibited a high degree of branching.

Visual observations and documentation revealed distinct variations in leaf hair coverage among different genotypes, with 16 genotypes displaying low (sparse) leaf hair density, 22 genotypes exhibiting moderate leaf hair density, and 4 genotypes showcasing a high level (dense) of leaf hair density.

The examination of various genotypes revealed that there were 10 genotypes exhibiting a light brown spikelet color, 10 genotypes with dark brown spikelets, 8 genotypes displaying a light purple spikelet color, 8 genotypes showing a dark purple spikelet color, and 6 genotypes presenting a creamy white appearance.

Following a visual examination, it was determined that 16 genotypes displayed minimal susceptibility to the disease, while another 16 genotypes showed a lower level of susceptibility. Additionally, seven genotypes were moderately affected, while two were highly affected and another two were extremely affected by the disease. Finally, one genotype exhibited a particularly high susceptibility to the blast disease.

The maximum similarity coefficient (1.00) of genotypes 1.RDC-1 and 2.RDC-1b is based on morphological data indicating that these two genotypes may share a similar gene pool. The lowest (0.00) similarity coefficient was observed between genotypes 1.RDC-1 and 30.RDC-24; in addition, the same similarity coefficient was recorded between the following pairs of genotypes: 2.RDC-1b and 30.RDC-24, 6 and 17, 6 and 27, 6 and 37, 6 and 39, 7 and 36, 8 and 20, 10 and 34, 11 and 30, 12 and 31, 12 and 40, 17 and 19, 19 and 27, 19 and 37, 20 and 31, 21 and 30, 26 and 27, 27 and 33, 29 and 31, 30 and 34, 31 and 39, 33 and 37, 36 and 38, and 39 and 40 (**Table 7**).

**Table 7:** Similarity matrix of Deenanath grass genotypes computed with the SM coefficient based on morphological data.

	1	2	3	4	5	6	7	8	9	10	11	12	13	14	15	16	17	18	19	20	21	22	23	24	25	26	27	28	29	30	31	32	33	34	35	36	37	38	39	40	41	42
1	1.00																																									
2	**1.00**	1.00																																								
3	0.43	0.43	1.00																																							
4	0.43	0.43	0.43	1.00																																						
5	0.57	0.57	0.57	0.86	1.00																																					
6	0.29	0.29	0.43	0.43	0.29	1.00																																				
7	0.57	0.57	0.29	0.43	0.57	0.14	1.00																																			
8	0.57	0.57	0.71	0.29	0.43	0.43	0.14	1.00																																		
9	0.43	0.43	0.57	0.43	0.43	0.29	0.14	0.57	1.00																																	
10	0.14	0.14	0.43	0.57	0.43	0.29	0.43	0.14	0.14	1.00																																
11	0.86	0.86	0.57	0.57	0.71	0.43	0.71	0.43	0.43	0.29	1.00																															
12	0.29	0.29	0.29	0.43	0.29	0.29	0.57	0.14	0.29	0.29	0.43	1.00																														
13	0.14	0.14	0.57	0.29	0.29	0.43	0.29	0.29	0.43	0.29	0.29	0.43	1.00																													
14	0.29	0.29	0.57	0.71	0.57	0.43	0.29	0.43	0.57	0.43	0.43	0.57	0.43	1.00																												
15	0.43	0.43	0.57	0.57	0.71	0.43	0.43	0.43	0.43	0.29	0.57	0.29	0.29	0.71	1.00																											
16	0.29	0.29	0.71	0.43	0.57	0.43	0.29	0.71	0.43	0.29	0.43	0.29	0.43	0.71	0.71	1.00																										
17	0.29	0.29	0.43	0.29	0.43	*0.00*	0.43	0.43	0.57	0.29	0.29	0.29	0.14	0.57	0.57	0.57	1.00																									
18	0.57	0.57	0.57	0.43	0.57	0.14	0.29	0.71	0.57	0.29	0.43	0.29	0.29	0.57	0.57	0.57	0.57	1.00																								
19	0.57	0.57	0.57	0.57	0.43	0.57	0.43	0.29	0.29	0.57	0.71	0.43	0.43	0.43	0.29	0.29	*0.00*	0.14	1.00																							
20	0.29	0.29	0.14	0.71	0.57	0.43	0.57	*0.00*	0.14	0.57	0.43	0.43	0.29	0.43	0.29	0.14	0.29	0.14	0.43	1.00																						
21	0.57	0.57	0.29	0.71	0.71	0.29	0.57	0.14	0.43	0.29	0.71	0.43	0.43	0.43	0.43	0.29	0.14	0.29	0.57	0.57	1.00																					
22	0.43	0.43	0.43	0.71	0.57	0.43	0.43	0.29	0.43	0.29	0.57	0.71	0.43	0.71	0.43	0.43	0.29	0.43	0.57	0.57	0.71	1.00																				
23	0.29	0.29	0.57	0.71	0.57	0.43	0.43	0.43	0.57	0.43	0.43	0.71	0.57	0.86	0.57	0.57	0.43	0.57	0.43	0.43	0.43	0.71	1.00																			
24	0.29	0.29	0.29	0.71	0.71	0.14	0.43	0.14	0.43	0.43	0.43	0.43	0.29	0.43	0.43	0.29	0.29	0.29	0.29	0.57	0.71	0.43	0.43	1.00																		
25	0.14	0.14	0.71	0.43	0.29	0.43	0.29	0.43	0.43	0.71	0.29	0.43	0.43	0.57	0.29	0.43	0.43	0.29	0.57	0.43	0.14	0.43	0.57	0.29	1.00																	
26	0.57	0.57	0.57	0.43	0.57	0.29	0.71	0.29	0.14	0.43	0.71	0.43	0.57	0.29	0.43	0.43	0.14	0.29	0.71	0.29	0.57	0.43	0.43	0.29	0.29	1.00																
27	0.14	0.14	0.29	0.43	0.43	*0.00*	0.29	0.29	0.57	0.29	0.14	0.29	0.14	0.43	0.29	0.29	0.71	0.43	*0.00*	0.43	0.29	0.43	0.43	0.43	0.43	0.00	1.00															
28	0.43	0.43	0.43	0.43	0.29	0.29	0.14	0.43	0.29	0.29	0.29	0.43	0.43	0.43	0.14	0.29	0.14	0.43	0.57	0.29	0.43	0.71	0.43	0.14	0.43	0.43	0.29	1.00														
29	0.29	0.29	0.14	0.14	0.14	*0.00*	0.43	0.14	0.43	0.14	0.29	0.43	0.14	0.43	0.29	0.29	0.71	0.29	0.14	0.29	0.29	0.43	0.29	0.14	0.29	0.14	0.57	0.29	1.00													
30	*0.00*	*0.00*	0.29	0.29	0.14	0.29	0.14	0.14	0.29	0.57	*0.00*	0.14	0.14	0.43	0.43	0.29	0.57	0.14	0.29	0.29	*0.00*	0.14	0.29	0.14	0.57	0.14	0.43	0.29	0.43	1.00												
31	0.57	0.57	0.71	0.29	0.43	0.43	0.14	0.71	0.43	0.29	0.43	*0.00*	0.29	0.29	0.57	0.43	0.29	0.57	0.43	0.00	0.14	0.14	0.29	0.14	0.43	0.43	0.14	0.43	0.00	0.43	1.00											
32	0.57	0.57	0.29	0.43	0.57	0.14	0.86	0.14	0.14	0.57	0.71	0.43	0.14	0.29	0.43	0.29	0.43	0.43	0.43	0.57	0.57	0.43	0.29	0.43	0.29	0.57	0.29	0.14	0.43	0.14	0.14	1.00										
33	0.43	0.43	0.57	0.43	0.57	0.43	0.43	0.29	0.14	0.43	0.57	0.14	0.57	0.43	0.57	0.57	0.29	0.29	0.57	0.43	0.43	0.29	0.29	0.29	0.29	0.71	0.00	0.29	0.14	0.29	0.43	0.43	1.00									
34	0.71	0.71	0.43	0.29	0.43	0.14	0.57	0.57	0.43	*0.00*	0.57	0.57	0.43	0.43	0.43	0.43	0.43	0.71	0.29	0.14	0.43	0.57	0.57	0.14	0.14	0.57	0.29	0.57	0.43	0.00	0.43	0.43	0.29	1.00								
35	0.43	0.43	0.43	0.71	0.57	0.29	0.14	0.57	0.57	0.29	0.29	0.43	0.29	0.71	0.43	0.43	0.43	0.71	0.29	0.43	0.43	0.71	0.71	0.43	0.43	0.14	0.57	0.71	0.29	0.29	0.43	0.14	0.14	0.57	1.00							
36	0.29	0.29	0.43	0.14	0.14	0.29	*0.00*	0.43	0.43	0.29	0.14	0.14	0.71	0.29	0.14	0.29	0.14	0.57	0.29	0.14	0.29	0.29	0.29	0.14	0.29	0.29	0.14	0.57	0.14	0.14	0.43	0.14	0.43	0.43	0.43	1.00						
37	0.14	0.14	0.29	0.29	0.29	*0.00*	0.29	0.29	0.71	0.14	0.14	0.57	0.43	0.43	0.29	0.29	0.57	0.43	*0.00*	0.14	0.29	0.29	0.57	0.57	0.29	0.14	0.57	0.14	0.43	0.29	0.14	0.14	*0.00*	0.43	0.43	0.29	1.00					
38	0.29	0.29	0.29	0.86	0.71	0.29	0.57	0.14	0.29	0.71	0.43	0.43	0.14	0.57	0.43	0.29	0.43	0.29	0.43	0.86	0.57	0.57	0.57	0.71	0.57	0.29	0.57	0.29	0.29	0.43	0.14	0.57	0.29	0.14	0.57	*0.00*	0.29	1.00				
39	0.14	0.14	0.29	0.29	0.29	0.29	0.29	0.14	0.29	0.14	0.29	0.57	0.43	0.57	0.43	0.43	0.43	0.29	0.14	0.43	0.29	0.43	0.43	0.43	0.29	0.14	0.29	0.14	0.57	0.14	*0.00*	0.29	0.43	0.29	0.29	0.29	0.43	0.29	1.00			
40	0.29	0.29	0.43	0.43	0.43	0.14	0.14	0.29	0.57	0.57	0.29	*0.00*	0.43	0.29	0.29	0.29	0.29	0.43	0.43	0.14	0.43	0.14	0.29	0.43	0.29	0.43	0.29	0.29	0.14	0.43	0.43	0.29	0.43	0.14	0.29	0.57	0.43	0.29	*0.00*	1.00		
41	0.14	0.14	0.43	0.29	0.14	0.29	0.57	0.14	0.14	0.57	0.29	0.71	0.57	0.43	0.14	0.29	0.29	0.14	0.57	0.43	0.29	0.57	0.57	0.14	0.71	0.57	0.29	0.57	0.43	0.43	0.14	0.43	0.29	0.43	0.29	0.29	0.29	0.43	0.29	0.14	1.00	
42	0.29	0.29	0.57	0.71	0.57	0.43	0.29	0.43	0.57	0.57	0.43	0.57	0.43	0.86	0.57	0.57	0.43	0.71	0.43	0.43	0.43	0.71	0.86	0.43	0.57	0.29	0.43	0.43	0.29	0.29	0.29	0.43	0.29	0.43	0.71	0.43	0.43	0.57	0.43	0.43	0.43	1.00

Bold value indicate maximum value and Italic value indicate minimum value.

**Figure 4:**
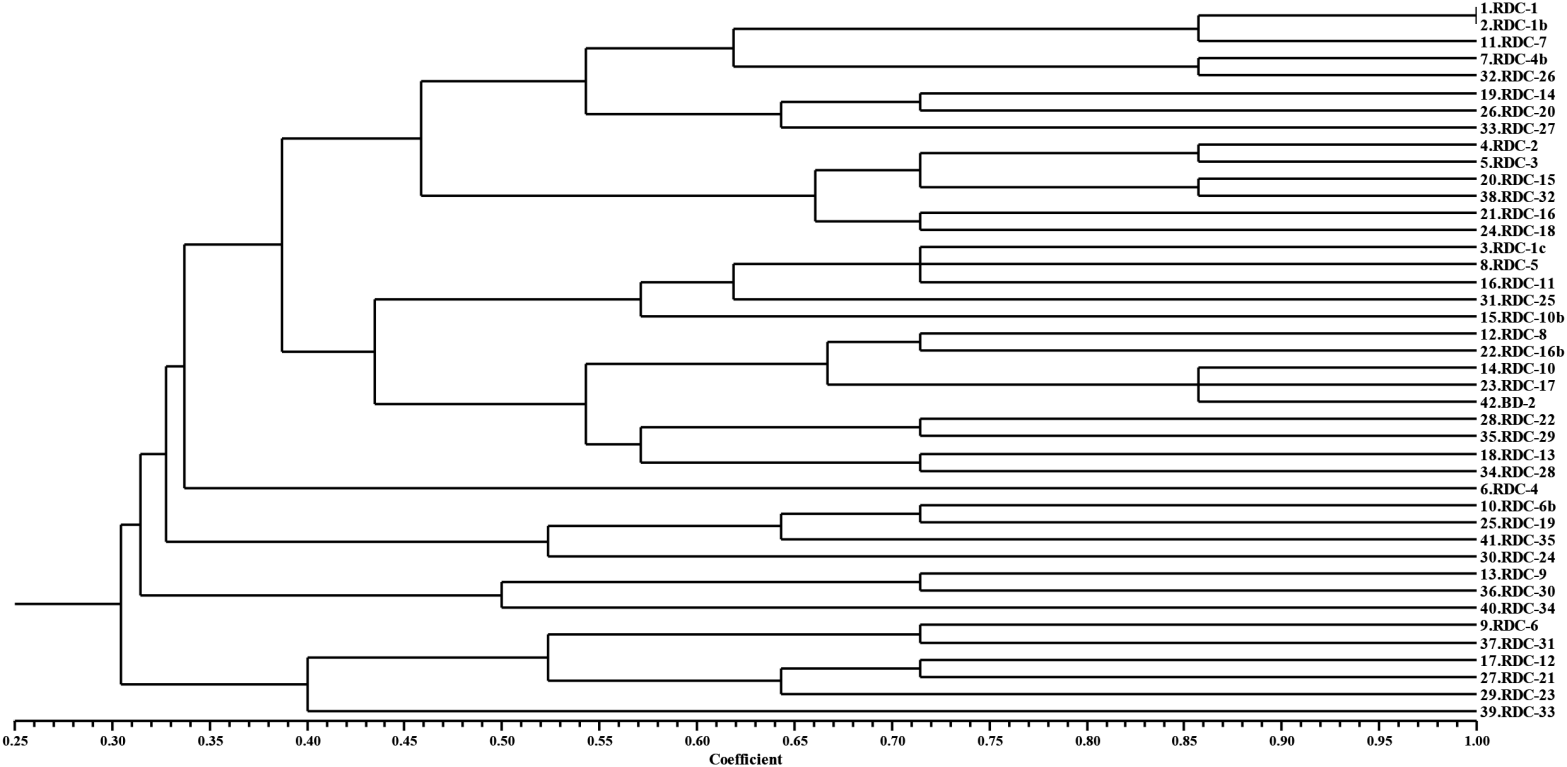
Hierarchical clustering based on morphological data for the 42 germplasm of Deenanath grass.

Jaccard’s similarity coefficients ranged between 0.30 and 0.86 for morphological based diversity. The dendrogram indicates that there was a major cluster “A” consisting of 36 genotypes. The other major cluster “B” consisted of six genotypes having a 0.30 similarity coefficient with cluster “A” (**Figure 4**).

The first cluster includes varieties RDC-1, RDC-1b, RDC-1c, RDC-2, RDC-3, RDC-4, RDC-4b, RDC-5, RDC-6b, RDC-7, RDC-8, RDC-9, RDC-10, RDC-10b, RDC-11, RDC-13, RDC-14, RDC-15, RDC-16, RDC-16b, RDC-17, RDC-18, RDC-19, RDC-20, RDC-22, RDC-24, RDC-25, RDC-26, RDC-27, RDC-28, RDC-29, RDC-30, RDC-32, RDC-34, RDC-35, and BD-2. While RDC-6, RDC-31, RDC-21, RDC-23, RDC-12, and RDC-33 fall in second cluster.

Major cluster “A” showed sub-clustering near the 0.32 similarity level. The two sub-clusters “A1” and “A2” consisted of 36 and 3 genotypes, respectively. Cluster A1 further showed sub-clustering near the 0.33 similarity levels as sub-cluster A1(a) and A1(b) and consisted of 29 and 4 genotypes, respectively. Cluster A2 further showed sub-clustering near the 0.50 similarity levels as sub-cluster A2(a) and A2(b) and consisted of two (RDC-9 and RDC-30) and one (RDC-34) genotype, respectively.

Major cluster “B” showed sub-clustering near the 0.40 similarity level. The two sub-clusters “B1” and “B2” consisted of five and one genotype, respectively. Cluster B1 further showed sub-clustering near the 0.54 similarity levels as sub-cluster B1(a) and B1(b) and consisted of two and three genotypes, respectively. Cluster B2 did not show sub-clustering and consisted of one genotype, RDC-33.

Genotypes RDC-1 and RDC-1b within cluster “A” are most similar with a similarity coefficient of 1.00, while in the lowest similarity index, i.e., the highest distance was between RDC-1 and RDC-33.

### Molecular characterization

#### PCR screening with SSR markers

**Figure 5:**
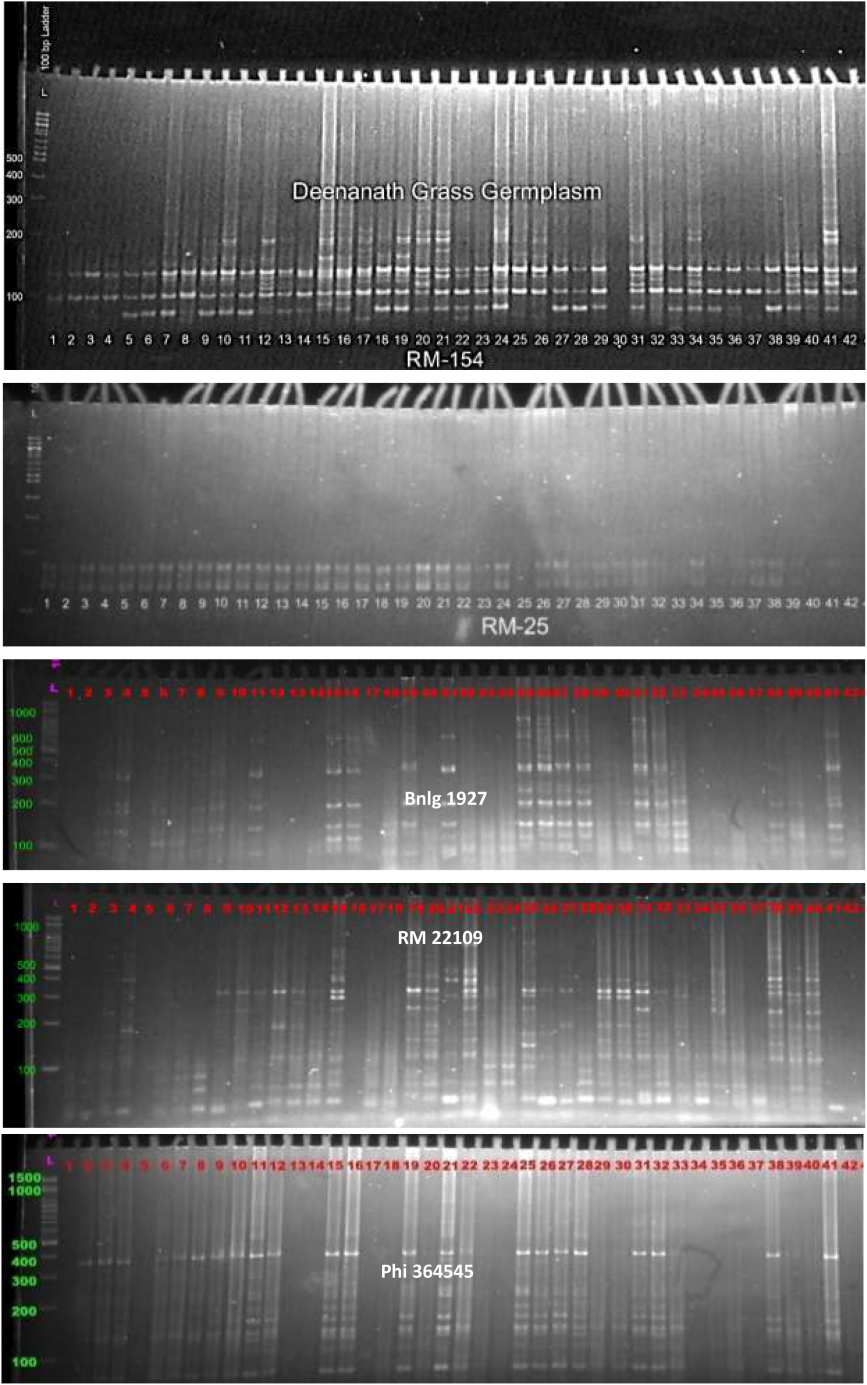
Amplification profile of Deenanath grass germplasm using SSR markers.

We used 17 random SSR markers in 42 Deenanath grass germplasm. Seventeen markers showed amplification, out of which 15 were amplified well and polymorphic. The average number of alleles per genotype per marker ranged from one to five. Here, the marker with the highest average number of alleles per genotype was bnlg 2228 with five alleles, while the markers with the lowest average number of alleles per genotype were RM 125 and RM 13, each with one allele. The PIC, calculated to estimate the discriminatory power of each marker, ranged from 0.38 for RM 26213 to 0.79 for bnlg 2228, with a mean PIC of 0.62. PIC value is a measure of the informativeness of a molecular marker as the basis for the classification and selection of markers that were efficient in discriminating among individuals. The highest EMR 24.33 was recorded with the marker/primer RM 21625, and its minimum EMR 0.00 was found with the marker/primer RM25, RM125, and RM13, with a mean EMR value of 14.83 per primer. The maximum MI was obtained with the marker/primer bnlg2228 (19.21), and the minimum was in the marker/primer RM25, RM125, and RM13 (0.00), with a mean MI value of 9.04. The resolving power indicates the discriminatory capability of the primers. The maximum Rp value was obtained with the marker/primer bnlg2228 (3.86), followed by RM 22109 (2.67) and bnlg 1927 (2.48), and the minimum value with RM25, RM125, and RM13 (0.00), with a mean Rp value of 1.56 for each primer. Therefore, the markers utilized here are very informative and can be recommended for molecular studies in Deenanath grass. The results are summarized in **Table 8**. **Figure 5** shows the PCR amplification profile of an SSR marker.

**Table 8:** Characteristics of polymorphic SSR markers obtained after the PCR screening of Deenanath grass genotypes.

S. no.	Marker	Band size	MB	PB	TB	PPB (%)	PIC	FPM	TBA	MR (*n*)	EMR	MI	Rp
1	phi 364545	160–370	1	2	3	66.67	0.66	0.67	82.00	27.33	18.22	12.05	2.10
2	bnlg 1927	120–300	0	3	3	100.00	0.66	1.00	66.00	22.00	22.00	14.45	2.48
3	bnlg 2109	190–600	1	2	3	66.67	0.61	0.67	51.00	17.00	11.33	6.90	1.86
4	bnlg 1194	110–210	0	3	3	100.00	0.63	1.00	69.00	23.00	23.00	14.55	2.05
5	RM25	140–180	2	0	2	0.00	0.50	0.00	84.00	42.00	0.00	0.00	0.00
6	RM125	150	1	0	1	0.00	0.00	0.00	42.00	42.00	0.00	0.00	0.00
7	RM 154	70–190	2	2	4	50.00	0.74	0.50	134.00	33.50	16.75	12.47	1.62
8	RM 277	120–150	1	1	2	50.00	0.49	0.50	53.00	26.50	13.25	6.51	1.48
9	RM 28153	180–220	3	0	3	0.00	0.67	0.00	105.00	35.00	0.00	0.00	1.00
10	RM 26213	50–200	0	2	2	100.00	0.38	1.00	44.00	22.00	22.00	8.25	0.95
11	RM 13	130	1	0	1	0.00	0.00	0.00	42.00	42.00	0.00	0.00	0.00
12	RM 22109	100–300	0	4	4	100.00	0.72	1.00	90.00	22.50	22.50	16.28	2.67
13	RM 21625	140–500	0	3	3	100.00	0.64	1.00	73.00	24.33	24.33	15.62	1.95
14	RM 307	120–400	1	1	2	50.00	0.41	0.50	45.00	22.50	11.25	4.62	1.10
15	RM 6054	60–220	0	3	3	100.00	0.62	1.00	64.00	21.33	21.33	13.28	2.10
16	bnlg2228	130–370	0	5	5	100.00	0.79	1.00	121.00	24.20	24.20	19.21	3.86
17	bnlg1176a	170–280	0	2	2	100.00	0.43	1.00	44.00	22.00	22.00	9.55	1.24
Sum			13.00	33.00	46.00	1,083.33	8.96	10.83	1,209.00	469.20	252.17	153.75	26.46
Average			0.76	1.94	2.71	63.73	0.53	0.64	71.12	27.60	14.83	9.04	1.56

Number of monomorphic bands (MB), number of polymorphic bands (PB), total number of bands (TB), percent of polymorphic bands (PPB), polymorphic information content (PIC), fraction of polymorphic markers (FPM), total band amplified (TBA), multiplex ratio (MR), effective multiplex ratio (EMR), marker index (MI), and resolving power (Rp).

### Population structure analysis

**Figure 6:**
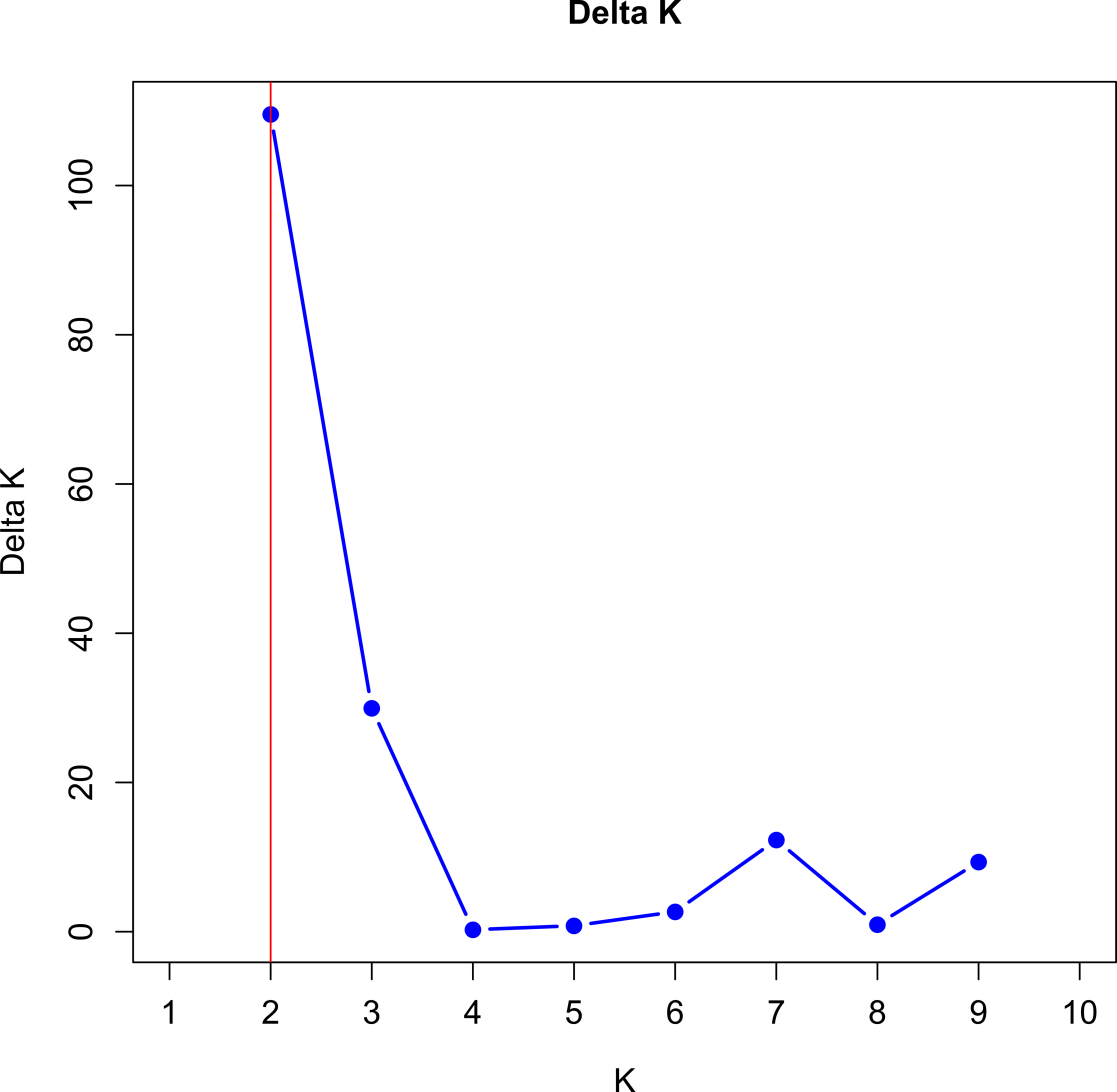
Delta *K* plot for population selection (*k* = 2).

The software STRUCTURE v 2.3.4 was used to estimate the genetic diversity of the rice accessions based on Δ*K*, and the highest peak was obtained at *k* =2 (**Figure 6**). Bayesian cluster-based STRUCTURE modelling analysis categorized the genotypes into two subpopulations, POP1 and POP2, corresponding to the wild type and the cultivated type.

**Figure 7:**
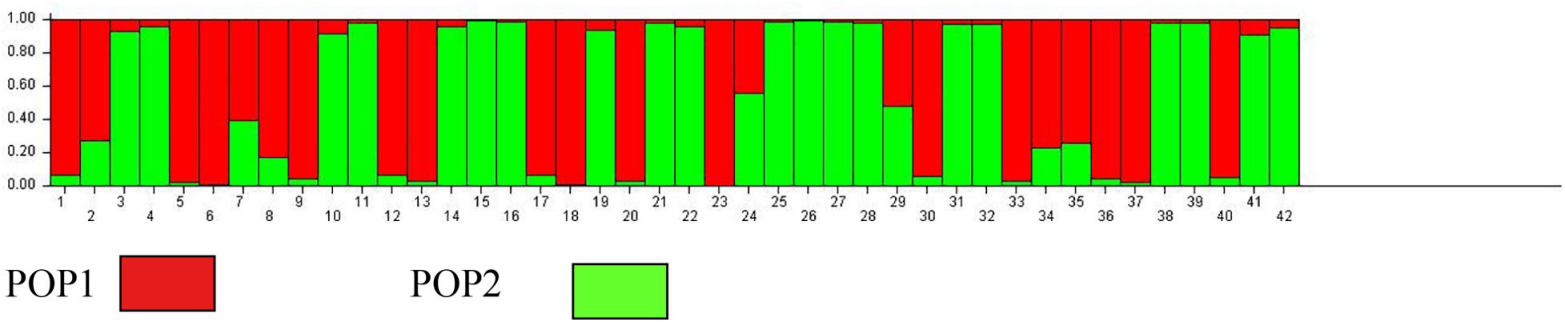
Bar plot showing genetic pools/diversity of 42 diverse Deenanath grass genotypes using 17 microsatellite (SSR) markers.

Out of 42 genotypes, 3 genotypes (Sr. nos. 23, 6, and 18) are under population 1, 2 genotypes (Sr. nos. 15 and 26) are under population 2, 12 genotypes (Sr. nos. 5, 37, 33, 13, 20, 9, 36, 40, 30, 1, 17, and 12) are mostly population 1 type, and 18 genotypes (Sr. no. 27, 16, 25, 21, 11, 38, 28, 39, 31, 32, 4, 14, 22, 42, 19, 3, 10, and 41) are mostly population 2 type. The diversity between the genotypes is ideal for selecting better genotypes when performing the experiments. The 5 genotypes show a pure line, while the remaining 37 genotypes show an admixture (**Figure 7**).

### Genetic similarity analysis

In addition to the allelic variation at the marker level, genetic similarity is an important and useful information that helps us to understand genetic relatedness and diversity and to find duplicates if any. In this study, a similarity matrix generated based on the Jaccard index using the SIMQUAL module in NTSYSpc was used for sequential, agglomerative, hierarchical clustering.

The 26.RDC-20 and 27.RDC-21 genotypes had the maximum genetic similarity coefficient (0.87), indicating that these genotypes may share a common gene pool (**Table 9**). The lowest (0.22) genetic similarity coefficient was observed between genotypes 1.RDC-1 and 15.RDC-10b, indicating that these two genotypes differ significantly at the genomic level.

**Table 9:** Similarity matrix of Deenanath grass genotypes computed with the SM coefficient based on SSR primers.

	1	2	3	4	5	6	7	8	9	10	11	12	13	14	15	16	17	18	19	20	21	22	23	24	25	26	27	28	29	30	31	32	33	34	35	36	37	38	39	40	41	42
1	1.00																																									
2	0.57	1.00																																								
3	0.35	0.63	1.00																																							
4	0.43	0.70	0.85	1.00																																						
5	0.67	0.57	0.30	0.39	1.00																																					
6	0.35	0.61	0.63	0.67	0.50	1.00																																				
7	0.39	0.67	0.78	0.83	0.43	0.85	1.00																																			
8	0.35	0.61	0.72	0.74	0.30	0.67	0.78	1.00																																		
9	0.35	0.63	0.63	0.65	0.39	0.80	0.74	0.72	1.00																																	
10	0.52	0.50	0.61	0.67	0.57	0.65	0.70	0.63	0.67	1.00																																
11	0.35	0.63	0.78	0.76	0.37	0.70	0.83	0.63	0.63	0.61	1.00																															
12	0.37	0.46	0.54	0.59	0.43	0.72	0.65	0.52	0.63	0.63	0.63	1.00																														
13	0.48	0.33	0.33	0.37	0.54	0.48	0.41	0.33	0.41	0.43	0.37	0.59	1.00																													
14	0.61	0.35	0.30	0.37	0.67	0.48	0.43	0.43	0.48	0.65	0.41	0.41	0.70	1.00																												
15	*0.22*	0.48	0.63	0.76	0.37	0.54	0.67	0.61	0.61	0.63	0.65	0.57	0.52	0.43	1.00																											
16	0.41	0.65	0.59	0.67	0.46	0.54	0.63	0.59	0.65	0.61	0.70	0.63	0.30	0.41	0.70	1.00																										
17	0.59	0.41	0.35	0.37	0.50	0.35	0.39	0.41	0.43	0.48	0.33	0.48	0.52	0.48	0.41	0.43	1.00																									
18	0.37	0.48	0.30	0.35	0.59	0.54	0.43	0.50	0.57	0.46	0.35	0.41	0.63	0.61	0.54	0.39	0.67	1.00																								
19	0.24	0.52	0.63	0.65	0.41	0.63	0.65	0.61	0.63	0.57	0.65	0.57	0.43	0.50	0.72	0.59	0.28	0.48	1.00																							
20	0.39	0.41	0.54	0.61	0.46	0.57	0.54	0.63	0.59	0.72	0.54	0.59	0.39	0.50	0.52	0.52	0.43	0.39	0.59	1.00																						
21	0.26	0.54	0.59	0.67	0.43	0.65	0.72	0.70	0.67	0.61	0.63	0.54	0.41	0.54	0.76	0.65	0.39	0.54	0.83	0.46	1.00																					
22	0.37	0.59	0.74	0.74	0.41	0.54	0.65	0.61	0.54	0.67	0.76	0.65	0.41	0.43	0.65	0.65	0.39	0.30	0.54	0.63	0.54	1.00																				
23	0.35	0.67	0.57	0.61	0.50	0.85	0.74	0.65	0.74	0.57	0.63	0.61	0.43	0.48	0.43	0.50	0.33	0.57	0.57	0.59	0.54	0.57	1.00																			
24	0.39	0.61	0.63	0.61	0.41	0.52	0.65	0.72	0.61	0.57	0.63	0.46	0.35	0.46	0.52	0.63	0.41	0.46	0.54	0.59	0.54	0.61	0.63	1.00																		
25	0.28	0.54	0.67	0.74	0.41	0.57	0.70	0.63	0.63	0.63	0.65	0.54	0.35	0.48	0.76	0.67	0.35	0.41	0.74	0.54	0.78	0.65	0.59	0.67	1.00																	
26	0.35	0.61	0.70	0.76	0.30	0.59	0.72	0.70	0.65	0.65	0.72	0.65	0.33	0.46	0.70	0.78	0.50	0.39	0.67	0.57	0.76	0.70	0.57	0.65	0.85	1.00																
27	0.30	0.54	0.65	0.76	0.35	0.63	0.76	0.65	0.61	0.63	0.80	0.70	0.35	0.43	0.74	0.76	0.39	0.37	0.72	0.57	0.76	0.74	0.57	0.57	0.76	**0.87**	1.00															
28	0.30	0.57	0.76	0.74	0.37	0.67	0.80	0.70	0.61	0.61	0.85	0.65	0.35	0.37	0.67	0.72	0.39	0.37	0.59	0.48	0.74	0.74	0.57	0.61	0.67	0.78	0.83	1.00														
29	0.37	0.54	0.61	0.65	0.46	0.57	0.63	0.65	0.50	0.67	0.65	0.57	0.30	0.50	0.57	0.63	0.35	0.39	0.63	0.67	0.54	0.65	0.67	0.61	0.59	0.70	0.74	0.65	1.00													
30	0.28	0.48	0.43	0.57	0.37	0.54	0.54	0.52	0.50	0.46	0.54	0.57	0.37	0.39	0.54	0.52	0.33	0.41	0.63	0.54	0.61	0.52	0.61	0.48	0.63	0.65	0.61	0.52	0.61	1.00												
31	0.20	0.46	0.67	0.70	0.33	0.50	0.63	0.61	0.57	0.54	0.72	0.52	0.52	0.37	0.85	0.57	0.37	0.48	0.63	0.52	0.65	0.67	0.43	0.52	0.72	0.65	0.65	0.74	0.52	0.54	1.00											
32	0.30	0.59	0.65	0.70	0.30	0.61	0.70	0.61	0.63	0.61	0.70	0.63	0.46	0.39	0.72	0.72	0.59	0.50	0.59	0.52	0.61	0.59	0.59	0.57	0.70	0.85	0.72	0.72	0.67	0.59	0.72	1.00										
33	0.33	0.59	0.57	0.67	0.46	0.87	0.76	0.67	0.80	0.59	0.67	0.74	0.48	0.48	0.59	0.61	0.41	0.59	0.67	0.59	0.65	0.54	0.76	0.43	0.52	0.63	0.72	0.70	0.65	0.59	0.57	0.65	1.00									
34	0.70	0.37	0.37	0.50	0.59	0.50	0.52	0.54	0.46	0.72	0.48	0.57	0.65	0.78	0.43	0.43	0.52	0.48	0.39	0.59	0.46	0.48	0.46	0.50	0.39	0.48	0.50	0.52	0.59	0.39	0.41	0.46	0.54	1.00								
35	0.63	0.37	0.43	0.48	0.63	0.54	0.57	0.43	0.50	0.63	0.48	0.57	0.67	0.76	0.50	0.41	0.39	0.41	0.46	0.50	0.48	0.52	0.48	0.52	0.52	0.46	0.46	0.52	0.52	0.46	0.50	0.43	0.50	0.76	1.00							
36	0.50	0.41	0.46	0.48	0.46	0.59	0.63	0.50	0.52	0.52	0.54	0.65	0.61	0.59	0.50	0.46	0.43	0.39	0.54	0.48	0.65	0.50	0.52	0.46	0.57	0.54	0.54	0.61	0.46	0.57	0.50	0.50	0.54	0.63	0.78	1.00						
37	0.52	0.37	0.54	0.41	0.54	0.46	0.41	0.43	0.39	0.54	0.41	0.43	0.63	0.72	0.37	0.28	0.33	0.39	0.43	0.46	0.43	0.52	0.46	0.30	0.39	0.37	0.35	0.46	0.52	0.37	0.41	0.33	0.43	0.61	0.67	0.61	1.00					
38	0.39	0.63	0.63	0.70	0.50	0.59	0.72	0.50	0.57	0.52	0.74	0.63	0.41	0.37	0.72	0.63	0.39	0.43	0.76	0.43	0.72	0.61	0.50	0.54	0.74	0.67	0.72	0.67	0.54	0.63	0.67	0.61	0.59	0.37	0.57	0.67	0.35	1.00				
39	0.46	0.37	0.52	0.48	0.28	0.48	0.54	0.52	0.50	0.48	0.63	0.61	0.52	0.52	0.46	0.59	0.46	0.33	0.57	0.50	0.48	0.48	0.43	0.52	0.52	0.63	0.65	0.57	0.57	0.43	0.48	0.59	0.54	0.57	0.48	0.59	0.48	0.57	1.00			
40	0.37	0.57	0.63	0.70	0.35	0.72	0.74	0.67	0.65	0.65	0.65	0.63	0.35	0.35	0.59	0.57	0.39	0.41	0.67	0.54	0.67	0.63	0.61	0.52	0.57	0.59	0.72	0.63	0.59	0.59	0.50	0.52	0.67	0.48	0.43	0.54	0.33	0.63	0.46	1.00		
41	0.35	0.57	0.61	0.61	0.48	0.57	0.65	0.67	0.65	0.59	0.61	0.57	0.35	0.41	0.70	0.74	0.35	0.46	0.76	0.57	0.74	0.48	0.48	0.54	0.72	0.65	0.67	0.63	0.61	0.54	0.65	0.61	0.63	0.43	0.48	0.59	0.39	0.67	0.54	0.61	1.00	
42	0.70	0.37	0.33	0.35	0.57	0.30	0.33	0.37	0.28	0.52	0.30	0.35	0.54	0.63	0.37	0.43	0.52	0.41	0.26	0.43	0.24	0.35	0.28	0.41	0.28	0.35	0.35	0.35	0.50	0.20	0.33	0.43	0.37	0.72	0.61	0.43	0.57	0.22	0.48	0.28	0.43	1.00

Bold value indicate maximum value and Italic value indicate minimum value.

**Figure 8:**
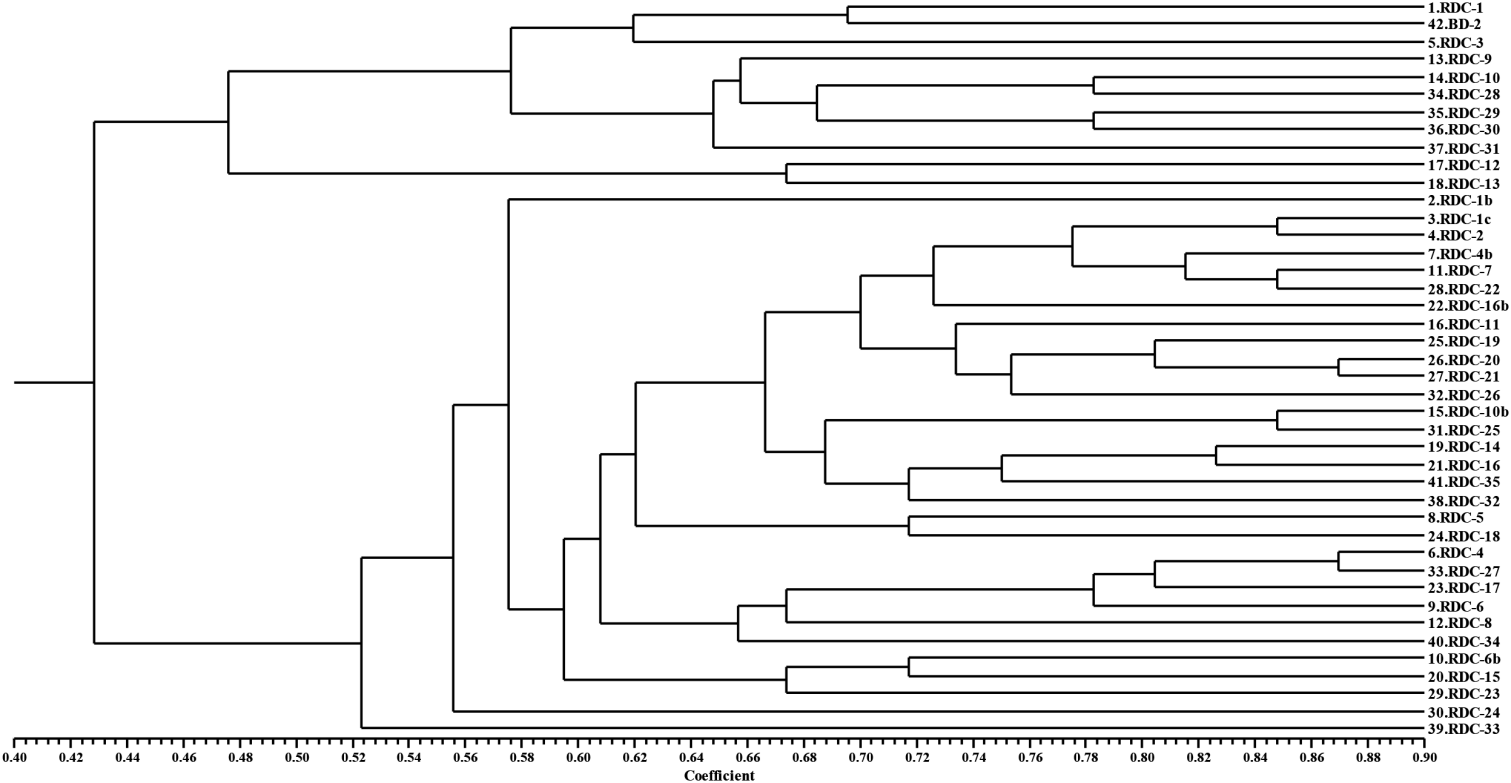
Hierarchical clustering based on molecular data for the 42 germplasm of Deenanath grass.

The dendrogram constructed grouped the genotypes into two clusters. Jaccard’s similarity coefficients ranged between 0.43 and 0.87 for molecular-based diversity. The dendrogram indicates that there was a major cluster “A” consisting of 11 genotypes. The other major cluster “B” consisted of 31 genotypes having a 0.43 similarity coefficient with cluster “A” (**Figure 8**).

Major cluster “A” showed sub-clustering near the 0.47 similarity level. The two sub-clusters “A1” and “A2” consisted of nine and two genotypes, respectively. Cluster A1 further showed sub-clustering near the 0.57 similarity levels as sub-cluster A1(a) and A1(b) and consisted of three and six genotypes, respectively. Cluster A2 showed sub-clustering near the 0.67 similarity levels and consisted of two genotypes RDC-12 and RDC-13.

Major cluster “B” showed sub-clustering near the 0.52 similarity level. The two sub-clusters “B1” and “B2” consisted of 30 and 1 genotypes, respectively. Cluster B1 further showed sub-clustering near the 0.55 similarity levels as sub-cluster B1(a) and B1(b) and consisted of 29 and 1 genotypes, respectively. Cluster B2 did not show sub-clustering and consisted of one genotype, RDC-33.

The highest similarity coefficient of 0.87 was observed between genotypes RDC-4 and RDC-27; RDC-20 and RDC-21 are within cluster “B”. The lowest similarity index, i.e., highest distance, was between RDC-1 and RDC-33.

## Discussion

In the present investigation, *D*^2^ analysis revealed the presence of significant diversity in the set of 42 germplasm assessed for the study. The germplasm were observed to be distributed in six different clusters. Similarly Mal et al. (1980) evaluated 36 P*. pedicellatum* accessions in a replicated trial for fodder yield and its attributes. The genotypic coefficient of variation was comparatively high for tiller number, culm thickness, leaf number, and fodder yield. Multivariate analysis, using the Mahalanobis *D*^2^ statistic, grouped the material into a pattern of nine varietal clusters, which differed from the geographical distribution pattern. Similar results were observed by Islam et al. (2020), as they used 30 maize inbred genotypes for *D*^2^ analysis and observed that these genotypes were grouped into seven clusters.

The main objective of cluster formation and assessing intra- and inter-cluster divergence is to facilitate the selection of suitable parents for a hybridization program. The underlying principle behind this grouping approach is that genotypes within the same cluster are expected to have less diversity among themselves compared to those in different clusters (Rao, 1952). Crossing genotypes within the same cluster is unlikely to yield the desired heterotic response. Therefore, when choosing parents for crossbreeding, it is important to select individuals from different clusters. The wider the inter-cluster difference and the greater the genetic diversity within the genotypes in those clusters, the better. Hence, when deciding on the parents for hybridization, factors such as genetic diversity, individual performance, and cluster mean for the specific trait being improved should all be considered.

In the current study, a total of 42 genotypes were analyzed and classified into six clusters. Previous research by Mehndiratta and Sidhy (1972) examined 36 forage sorghum genotypes, which were categorized into nine clusters, while Umakanth et al. (2002) studied 48 forage sorghum genotypes and identified five clusters.

The clustering pattern observed in this study confirms that genetic diversity does not necessarily align with geographic diversity. In some cases, genotypes originating from different geographical areas were grouped together, while different genotypes collected from the same area were placed in separate clusters. Similar findings were also reported by Mehndiratta and Sidhy (1972) and Umakanth et al. (2002).

Results of agro-morphological characterization revealed that all quantitative and qualitative traits discussed in the study are tools for estimating the genetic diversity of this study. Various metric measurements and visual observations are part of characterization and analysis that are necessary for Deenanath grass assortment and assessment. However, morphological characterization is subject to environmental effects, and it is the first step in the explanation and cataloguing of the germplasm. These outcomes conform with findings by Singh et al. (2021); Mishra and Katiyar (1990); Asmare et al. (2017); Gadisa et al. (2019); Heliso et al. (2019), and Singh et al. (2020).

Knowledge of genetic diversity is important for successful genetic improvement program. Genetic diversity between populations indicates the differences in gene frequencies. In addition to estimates of variability, knowledge of genetic diversity among genotypes is essential for selecting diverse parents for the hybridization program. PIC, calculated to estimate the discriminatory power of each marker, ranged from 0.00 for RM 125 and RM 13 to 0.79 for bnlg 2228, with a mean PIC of 0.53. Similarity coefficients ranged between 0.43 and 0.87 for molecular-based diversity. The dendrogram indicates that there was a major cluster “A” consisting of 11 genotypes and a major cluster “B” consisting of 31 genotypes at 0.43 similarity coefficient.

Similarly, Saxena and Chandra (2011) used sequence-tagged site (STS) markers to find out genetic linkages among marvel grass (*Dichanthium annulatum* Forsk.) accessions. In total, 17 *Stylosanthes*-derived STS primers were evaluated for reactivity with 30 accessions of *Dichanthium annulatum*. Fourteen (82.4%) of these responded, yielding a total of 106 (84 polymorphic) bands. Individual primer pair bands varied from 4 to 11 with an average of 7.57 bands, whereas polymorphic bands ranged from 4 to 9 with an average of 6.0 bands, resulting in an average polymorphism of 80.1%. PIC ranged from 0.22 to 0.50, whereas the MI ranged from 1.33 to 4.49.

Based on morphological data, genotypes RDC-1 and RDC-1b within cluster “A” are the most similar with a similarity coefficient of 1.00, indicating that these two genotypes may share a similar gene pool, while the lowest similarity index, i.e., the highest distance, was between RDC-1 and RDC-33, 1.RDC-1 and 30.RDC-24, 2.RDC-1b and 30.RDC-24, 6 and 17, 6 and 27, 6 and 37, 6 and 39, 7 and 36, 8 and 20, 10 and 34, 11 and 30, 12 and 31, 12 and 40, 17 and 19, 19 and 27, 19 and 37, 20 and 31, 21 and 30, 26 and 27, 27 and 33, 29 and 31, 30 and 34, 31 and 39, 33 and 37, 36 and 38, and 39 and 40, indicating that they differ significantly at the genomic level and can be used to develop bi-parental mapping populations as well as to broaden the genetic background.

Based on molecular diversity, genotypes 26.RDC-20 and 27.RDC-21 had the maximum genetic similarity coefficient (0.87), indicating that these genotypes may share a common gene pool. The lowest (0.22) genetic similarity coefficient was observed between genotypes 1.RDC-1 and 15.RDC-10b, which indicates that these two genotypes differ significantly at the genomic level and can be used to develop bi-parental mapping populations as well as broaden the genetic base.

Morphological markers do not discriminate genotypes at such a level; hence, many genotypes have the lowest similarity coefficient. On the other hand, molecular markers are more powerful than morphological markers in terms of discriminating genotypes.

The integration of morphological characterization, molecular marker diversity, and population structure analysis provides a comprehensive view of genetic variability in Deenanath grass. STRUCTURE analysis revealed two major sub-populations (POP1: wild type, POP2: cultivated type), with most genotypes showing admixture. Morphological clustering (Mahalanobis *D*² and Jaccard’s similarity) highlighted distinct trait associations across clusters, while SSR marker analysis confirmed substantial allelic diversity and informative PIC values.

A clear correlation emerges between population structure and morphological traits. POP1 genotypes generally aligned with clusters exhibiting early maturity, shorter plant height, and lower biomass, consistent with wild-type characteristics. In contrast, POP2 genotypes overlapped with clusters showing higher green fodder yield (GFY), dry matter yield (DMY), crude protein content, and larger leaf dimensions, reflecting cultivated type. For example, cluster III, which displayed superior values for plant height, GFY, DMY, and crude protein, corresponded largely to POP2 genotypes, while cluster II, characterized by early flowering and lower moisture content, overlapped with POP1 genotypes.

Within these groups, specific superior cultivated-type genotypes can be highlighted. RDC-20 and RDC-21 (POP2, cluster III) exhibited high GFY and crude protein yield, supported by strong molecular similarity (0.87), making them promising candidates for forage yield and nutritional quality. Conversely, wild-type genotypes RDC-6 and RDC-18 (POP1, cluster II) combined early maturity with disease tolerance, offering resilience under stress conditions. Admixture lines such as RDC-12 and RDC-13, positioned between POP1 and POP2, represent valuable bridging material for combining early maturity with high biomass.

## Conclusion

The maximum PIC and Rp value was obtained with the markers/primers bnlg2228, RM 22109, and bnlg 1927, which means it is very informative and can be further used for molecular studies in Deenanath grass. The most diverse genotypes 1.RDC-1 and 15.RDC-10b based on similarity index indicate that these two genotypes differ significantly at the genomic level and can be used in crop improvement by broadening the genetic base. Genotypes 26.RDC-20 and 27.RDC-21 had the maximum genetic similarity coefficient, indicating that these genotypes may share a common gene pool and not attempt crosses between them. We discovered new and divergent variants by inferring the molecular genetic similarity of renowned enhanced germplasm of Deenanath grass. We also looked at the primary morphological characteristics of improved germplasm to see if they matched the genetic similarity found. We discover that genotype grouping based on molecular and morphological data follows a similar pattern. This could imply that the majority of physically identical types share the same genetic background at the DNA level. The findings of this study can be used to create breeding program that select genetically different parents from among released variants. Understanding the variance and similarity in improved varieties provides insights into agricultural improvement status and can spark more in-depth research into crop improvement and genetic erosion dynamics.

By integrating morphological, molecular, and population structure analyses, this study identifies clear correlations between genetic sub-populations and trait performance. POP2 genotypes (cultivated type) are associated with superior yield and nutritional traits, while POP1 genotypes (wild type) contribute early maturity and stress resilience. Admixture lines provide opportunities to represent valuable bridging material for trait introgression. Genotypes such as RDC-20, RDC-21, and RDC-6 are recommended as promising parental material for future breeding programs aimed at enhancing both productivity and adaptability in Deenanath grass.

## Data Availability

The raw data supporting the conclusions of this article will be made available by the authors, without undue reservation.

## References

[B1] Anandaraj M. Prasath K. Kandiannan T. John Zachariah V. Srinivasan A. K. Jha B. K. . (2014). Genotype by environment interaction effects on yield and curcumin in turmeric (Curcuma longa L.). Ind. Crops Prod. 53, 358–364. doi: 10.1016/j.indcrop.2014.01.005. PMID: 38826717

[B2] Asmare B. Demeke T. Tolemariam F. Tegegne A. Haile Wamatu J. (2017). Effects of altitude and harvesting dates on morphological characteristics, yield and nutritive value of desho grass (Pennisetum pedicellatum Trin.) in Ethiopia. Agric. Nat. Resour. 51, 148–153. doi: 10.1016/j.anres.2016.11.001. PMID: 38826717

[B3] Bhandari H. R. Bhanu A. N. Srivastava K. Singh M. N. Shreya H. A. (2017). Assessment of genetic diversity in crop plants-an overview. Adv. Plants Agric. Res. 7, 279–286. doi: 10.15406/apar.2017.07.00255

[B4] Botstein D. White R. L. Skalnick M. H. Davies R. W. (1980). Construction of a genetic linkage map in man using restriction fragment length polymorphism. Am. J. Hum. Genet. 32, 314–331 6247908 PMC1686077

[B5] Doyle J. J. Doyle J. L. (1987). A rapid DNA isolation procedure for small quantities of fresh leaf tissues. Phytochem. Bull. 19, 11–15.

[B6] Gadisa B. Dinkale T. Debela M. (2019). Evaluation of desho grass (Pennisetum pedicellatum Trin) lines for their adaptability at Mechara Research station, Eastern Oromia, Ethiopia. J. Ecol. Natural Environ. 11, 26–32. doi: 10.5897/JENE2019.0742

[B7] Heliso M. F. Hibebo D. K. Atumo T. T. Tunkala B. Z. Dula M. G. (2019). Evaluation of desho grass (Pennisetum pedicellatum) productivity under different fertilizer combinations and spacing at Gamo Gofa zone, Ethiopia. J. Agric. Environ. Sci. 4, 50–59. Available online at: https://orcid.org/0000-0001-6347-7058.

[B8] Islam N. U. Ali G. Dar Z. A. Maqbool S. Kumar B. Bhat A. (2020). Genetic divergence in maize (Zea mays L.) inbred lines. Int. J. Chem. Stud. 8, 425–428. doi: 10.22271/chemi.2020.v8.i1f.8284

[B9] Jarvis A. Lane A. Hijmans R. J. (2008). The effect of climate change on crop wild relatives. Agric. Ecosyst. Environ. 126, 13–23. doi: 10.1016/j.agee.2008.01.013. PMID: 38826717

[B10] Khan A. F. Paramathma M. Amirthadevarathinam A. Sivasamy N. Sudhakar D. Bose M. S. C. (1995). Deenanath Co-1: a new annual fodder grass for Tamil Nadu. Madras Agric. J. 82, 510–511. doi: 10.29321/maj.10.a01247

[B11] Mahalanobis P. C. (1928). On the need for standardisation in measurements on the living. Biometrika 20, 1–31. doi: 10.2307/2331938

[B12] Mal B. Patil B. D. Yadav M. S. (1980). “ Gene pool and its classification in dinanath grass,” in Trends in genetical research on pennisetums, 275–278.

[B13] Mehndiratta P. D. Sidhy B. S. (1972). Studies on genetic diversity in forage Sorghum. Plant Sci. India 4, 16–20.

[B14] Mishra V. S. Katiyar D. S. (1990). Dinanath grass: a promising fodder for tropical and sub-tropical regions. Indian Farming 40, 17–18.

[B15] Mukherjee A. K. Roquib M. A. Bandopadhyay S. K. Mandal B. B. (1982). Review of research on deenanath grass (Pennisetum pedicellatum Trin.). Forage Res. 8, 11–17.

[B16] Nadeem M. A. Nawaz M. A. Shahid M. Q. Doğan Y. Comertpay G. Yıldız R. . (2018). DNA molecular markers in plant breeding: current status and recent advancements in genomic selection and genome editing. Biotechnol. Biotechnol. Equip. 32, 261–285. doi: 10.1080/13102818.2017.1400401. PMID: 37339054

[B17] Prevost A. Wilkinson M. J. (1999). A new system of comparing PCR primers applied to ISSR fingerprinting of potato cultivars. Theor. Appl. Genet. 98, 107–112. doi: 10.1007/s001220051046. PMID: 30311153

[B18] Pritchard J. K. Stephens M. Donnelly P. (2000). Inference of population structure using multilocus genotype data. Genetics 155, 945–959. doi: 10.1093/genetics/155.2.945 10835412 PMC1461096

[B19] Rao C. R. (1952). Advanced statistical methods in Biometrical research (New York: John Willey and Sons. Ins). doi: 10.2307/1417958

[B20] Rohlf F. J. (2000). NTSYS-pc: Numerical taxonomy and multivariate analysis system, version 2.10e (New York: Exeter Publications).

[B21] Saxena R. Chandra A. (2011). Transferability of STS markers in studying genetic relationships of marvel grass (Dichanthium annulatum). J. Environ. Biol. 32, 701–706. 22471204

[B22] Singh S. D. Navi S. S. (2000). Genetic resistance to pearl millet downy mildew II. Resistance in wild relatives. J. Mycology Plant Pathol. 30, 167–171. doi: 10.1094/pd-79-0545

[B23] Singh S. K. Kautkar S. Gurjar B. Pathak P. K. Swami S. (2020). Engineering properties of spikelets and true seeds of deenanath (Pennisetum pedicellatum Trin.) grass. Range Manage. Agrofor. 41, 328–335. Available online at: https://www.rmaj.in/rma/article/view/104.

[B24] Singh T. Dheeravathu S. N. Dikshit N. Manjunatha N. Sahay G. (2021). Collection and evaluation of genetic diversity in Dinanath grass (Pennisetum pedicellatum Trin.) for forage yield and leaf blight resistance. J. Environ. Biol. 42, 1355–1362. doi: 10.22438/jeb/42/5/MRN-1487

[B25] Umakanth A. V. Madhusudhana R. Swarnlata Kaul S. K. Rana B. S. (2002). Genetic diversity studies in sorghum. Int. Sorghum Millets News Lett. 43, 31–33.

[B26] Varshney R. K. Chabane K. Hendre P. S. Aggarwal R. K. Graner A. (2007). Comparative assessment of EST-SSR, EST-SNP and AFLP markers for evaluation of genetic diversity and conservation of genetic resources using wild, cultivated and elite barleys. Plant Sci. 173, 638–649. doi: 10.1016/j.plantsci.2007.08.010. PMID: 38826717

